# Genome-wide association study of developmental dysplasia of the hip identifies an association with *GDF5*

**DOI:** 10.1038/s42003-018-0052-4

**Published:** 2018-05-31

**Authors:** Konstantinos Hatzikotoulas, Andreas Roposch, The DDH Case Control Consortium, Andrew Wainwright, Tim Theologis, Nicholas M. P. Clarke, Jonathan S. M. Dwyer, Aresh Hashemi-Nejad, Nigel Kiely, Marcos Katchburian, Nicolas Nicolaou, Johnathan Page, Martin Gargan, Colin Bruce, Anish Sanghrajka, Paul Marshall, Mark Flowers, Olivia Malaga-Shaw, Piers Mitchell, Ben Holroyd, Manoj Ramachandran, Karan M. Shah, Matthew J. Clark, Selina Bratherton, Vasanti Limbani, Julia Steinberg, Eleni Zengini, Kaltuun Warsame, Madhushika Ratnayake, Maria Tselepi, Jeremy Schwartzentruber, John Loughlin, Deborah M. Eastwood, Eleftheria Zeggini, J. Mark Wilkinson

**Affiliations:** 1 Wellcome Trust Sanger Institute, Wellcome Trust Genome Campus, Morgan Building, Hinxton, Cambridge, CB10 1HH UK; 2 Institute of Child Health, University College London, 30 Guildford Street, London, WC1N 3EH UK; 3 Department of Oncology and Metabolism, University of Sheffield, Medical School, Beech Hill Road, Sheffield, S10 2RX UK; 4 Royal National Orthopaedic Hospital, Brockley Hill, Stanmore, Middlesex HA7 4LP UK; 5 Institute of Genetic Medicine, Newcastle University, Newcastle upon, Tyne, NE2 4HH UK; 6 Nuffield Orthopaedic Centre, Oxford, OX3 7HE UK; 7 Southampton General Hospital, Southampton, SO16 6YD UK; 8 University Hospital of the North Midlands, Stoke-on-Trent, ST4 6QG England; 9 Royal National Orthopaedic Hospital, Stanmore, HA7 4LP UK; 10 Robert Jones & Agnes Hunt Hospital, Oswestry, SY10 7AG UK; 11 Maidstone Hospital, Maidstone, ME16 9QQ UK; 12 County Durham & Darlington Hospital, Durham, DH1 5TW UK; 13 Royal Hospital for Children, Bristol, BS2 8BJ UK; 14 Alder Hey Children’s Hospital, Liverpool, L12 2AP UK; 15 Norfolk & Norwich University Hospital, Norwich, NR4 7UY UK; 16 Royal Lancaster Infirmary, Lancaster, LA1 4RO UK; 17 Children’s Hospital, Sheffield, S10 2TH UK; 18 Royal Free Hospital, London, NW3 2QG UK; 19 Peterborough & Stamford Hospital, Peterborough, PE3 9GZ UK; 20 Derriford Hospital, Plymouth, PL6 8DH UK; 21 Bart’s and The Royal London Children’s Hospital, London, E1 1BB UK

## Abstract

Developmental dysplasia of the hip (DDH) is the most common skeletal developmental disease. However, its genetic architecture is poorly understood. We conduct the largest DDH genome-wide association study to date and replicate our findings in independent cohorts. We find the heritable component of DDH attributable to common genetic variants to be 55% and distributed equally across the autosomal and X-chromosomes. We identify replicating evidence for association between *GDF5* promoter variation and DDH (rs143384, effect allele A, odds ratio 1.44, 95% confidence interval 1.34–1.56, *P* = 3.55 × 10^−22^). Gene-based analysis implicates *GDF5* (*P* = 9.24 × 10^−12^), *UQCC1* (*P* = 1.86 × 10^−^^10^), *MMP24* (*P* = 3.18 × 10^−9^), *RETSAT* (*P* = 3.70 × 10^−^^8^) and *PDRG1* (*P* = 1.06 × 10^−^^7^) in DDH susceptibility. We find shared genetic architecture between DDH and hip osteoarthritis, but no predictive power of osteoarthritis polygenic risk score on DDH status, underscoring the complex nature of the two traits. We report a scalable, time-efficient recruitment strategy and establish for the first time to our knowledge a robust DDH genetic association locus at *GDF5*.

## Abstract (web summary)

Konstantinos Hatzikotoulas et al. report the largest genome-wide association study to date for developmental dysplasia of the hip using national clinical audit data from the UK. They find a significant association with the *GDF5* locus and evidence for shared genetic architecture with hip osteoarthritis.

## Introduction

Developmental dysplasia of the hip (DDH) is a disorder characterised by abnormal development of the hip joint and presents with varying severity from mild uncovering of the femoral head to complete dislocation of the joint^1^. It is the most common developmental musculoskeletal anomaly, with population-weighted average incidence that ranges strongly with ethnic background from 0.06 per 1000 live births in Black Africans to 76.1 per 1000 live births in Native Americans^2^, and with an incidence in the UK European population of 3.6 per 1000. DDH is a complex disorder, with known associations including female sex, first-born, breech presentation, and family history^2^. There is a seven-fold increase in the incidence between siblings and a 10-fold increase in the parents of probands compared to the general population^3^, and a concordance rate of 41% between identical twins versus 3% in dizygotic twins^4^.

While DDH is heritable, its genetic architecture remains poorly characterised. Several linkage scans and candidate gene studies have implicated possible associated genetic variants, including in *GDF5*^5^, but to date no replicated loci of genome-wide significance have been identified^2,6–9^. A recent genome-wide association study (GWAS) of 386 patients and 558 controls in the Han Chinese population suggested an association with variation in *UQCC* (odds ratio (OR) 1.35, *P* = 3.63 × 10^-6^), a gene adjacent to *GDF5*^10^. Morphological abnormalities of the hip such as DDH are also recognised as risk factors for the development of secondary degenerative change of the hip^11,12^, and commonly result in hip replacement in adult life^12,13^. Idiopathic hip osteoarthritis is also very common and has a substantial heritable component^14^, and may share common genetic aetiology with DDH^2,7,15^.

Systematic examination of genome-wide variation in DDH in larger sample sizes is necessary to clarify its heritable biology and inform mechanism-driven preventative strategies. However, sample collection for uncommon diseases can be challenging and time-consuming, as attested by the relative paucity of sample sets for genetic studies of DDH. Routinely collected national clinical audit datasets present an opportunity for efficient case ascertainment in genetic epidemiology. The National Joint Registry for England, Wales, Northern Ireland, and the Isle of Man (NJR, http://www.njrcentre.org.uk/) was established in 2003 to collect audit data on all hip and knee replacement surgery in these regions, for which it has a completeness rate of 97% (http://www.njrreports.org.uk/Data-Completeness-and-quality). As at 31 December 2014, the dataset held information on 711,765 primary hip replacement procedures, including data on the diagnostic indication for surgery.

We use the NJR as a case-ascertainment tool to conduct a nationwide genome-wide association scan to characterise the genetic architecture of DDH. We examine the heritable contribution to DDH, and use fine-mapping and gene-based enrichment approaches to identify likely causal variants and genes. Finally, we use independent osteoarthritis datasets to examine the potential for shared genetic aetiology between DDH and hip osteoarthritis. We report a scalable, time-efficient recruitment strategy. We find the heritable component of DDH attributable to common variants to be 55% with the lead replicating signal at genome-wide significance being rs143384 within the *GDF5* promoter. We also find shared genetic architecture between DDH and hip osteoarthritis.

## Results

### Recruitment strategy

**Fig. 1:**
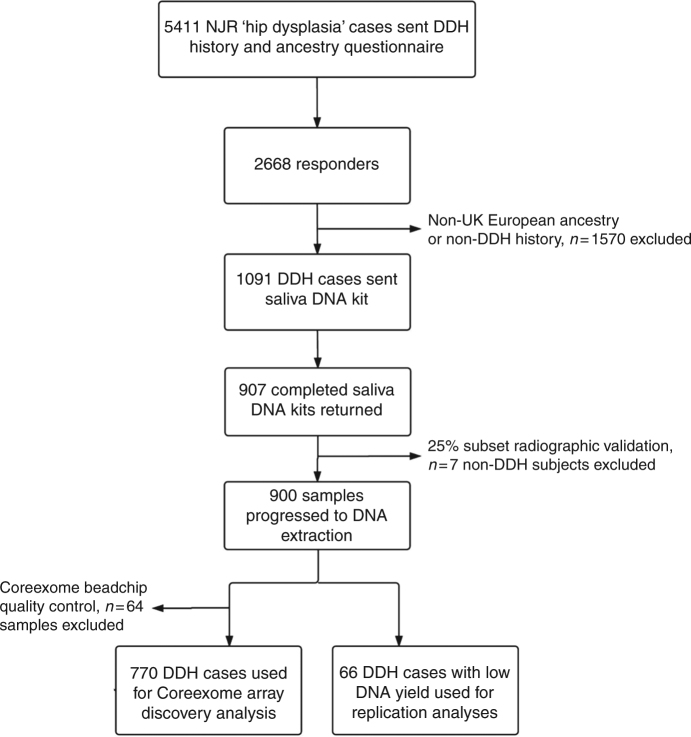
Recruitment flowchart

At the time of recruitment, the NJR had “hip dysplasia” recorded as the indication for hip replacement surgery in 5411 patients from the total database of 711,765 procedures (0.76%). All of these individuals were invited to participate in the postal screening phase of the study (Fig. 1). Following screening, 1091 consenting individuals with self-confirmed UK European ancestry and idiopathic DDH diagnosed in childhood were invited to submit a saliva sample. Of the 907 who returned a sample, 834 provided sufficient DNA to proceed to genome-wide genotyping. After quality control, 64 more individuals were excluded, leading the final number of DDH cases used in the genome-wide association analysis to 770. The sex distribution of the responding subjects was similar to that of the DDH population in the UK^4^. The diagnosis of DDH was independently validated by assessment of pelvic radiographs in a convenience sample of 25% of the subjects completing saliva return. The age and sex distribution of the radiographic validation subset was similar to the 907 that returned a saliva sample and radiographic analysis confirmed DDH in 224/231 (97%) subjects. Non-DDH cases were excluded from further analysis. The time taken from first patient mailshot to completion of genome-wide association analysis was approximately 18 months.

Controls were drawn from the United Kingdom Household Longitudinal Study (UKHLS), also known as Understanding Society (http://www.understandingsociety.ac.uk/). The UKHLS is an annual longitudinal panel survey of over 40,000 UK households from England, Scotland, Wales and Northern Ireland beginning in 2009–2010. Participants are surveyed annually and the study includes a wide range of sociodemographic, phenotypic, self-reported medical history and medication use data. Biomedical measures and blood samples were collected during a single nurse visit and DNA has been extracted and stored for genetic analyses. In total, 10,480 samples were genotyped on the Illumina HumanCoreExome-12v1-0_A chip at the Wellcome Trust Sanger Institute. After quality control (QC), genotype data for 9961 individuals were available. We further excluded all participants with any musculoskeletal disorder and the final dataset comprised 8016 individuals. A case:control ratio of ~1:4 was selected to be used in both discovery and replication stage to guard against case:control imbalance causing association tests to miscalculate for low frequency variants^16^.

### The heritable component of DDH

Using genetic complex trait analysis (GCTA)^17^ across 770 cases and 3364 controls in the discovery GWAS, we found that common-frequency autosomal single-nucleotide polymorphisms (SNPs) explain 55% (±se = 6%, *P* < 0.0001) of the liability-scale heritability of DDH (Supplementary Table 1). The heritability estimate was similar (54.7 ± 5.8%) when the analysis was repeated using sex as a covariate. When the heritability analysis was stratified by chromosome, we found that the X chromosome contributed a similar amount to overall DDH heritability as each of the autosomes (Supplementary Fig. 1), and we identified no individual X-chromosome signals.

### Discovery GWAS

**Fig. 2:**
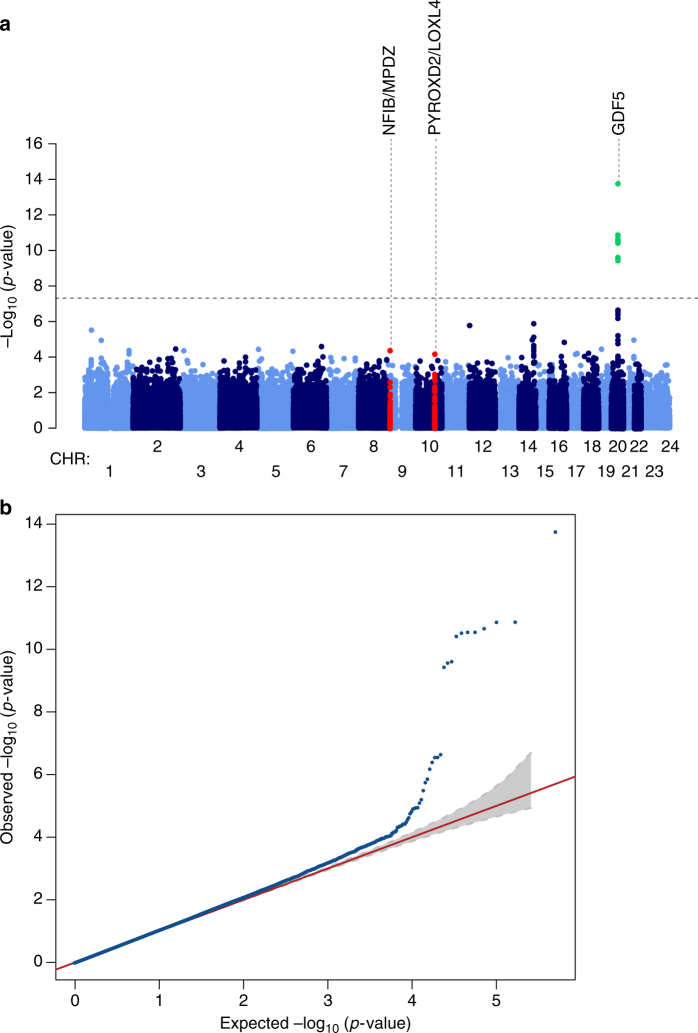
Manhattan and quantile–quantile plot of the DDH genome-wide association scan. **a** The dashed line indicates the genome-wide significance threshold (*P* = 5.0 × 10^-8^). Green dots represent variants for which *P*-values reached the genome-wide significance threshold and red dots illustrate the other two replicating signals. Chromosomes X and pseudo-autosomal regions on the chromosome X are represented by number 23 and 24, respectively. **b** Quantile–quantile plot of the data used in the GWAS

Genome-wide likelihood ratio test under an additive genetic model for association with DDH showed an excess of signals in the discovery sample set (Fig. 2a, b). Data were pruned for linkage disequilibrium using the clumping function in PLINK and 53 SNPs comprising 25 independent signals showed suggestive evidence for association with DDH with *P* < 1 × 10^−4^ compared to three independent variants expected under the null hypothesis of no association (binomial *P* = 9.57 × 10^−17^, Supplementary Table 2). Eleven correlated variants reached genome-wide significance (*P* < 5.0 × 10^−8^) and all reside in the same region. The lead variant, rs143384 (effect allele A, effect allele frequency (EAF) 0.60, OR [95% CI] 1.57 [1.3–1.77], *P* = 1.72 × 10^−14^), is located in the 5′ untranslated region (UTR) of *GDF5* (20q11.22). We performed a conditional analysis specifically for rs143384 and rs143383 (effect allele A, EAF 0.64, OR [95% CI] 1.51 [1.33–1.70], *P* = 1.29 × 10^−11^) as they have both previously shown suggestive association with DDH. When rs143383 is conditioned on rs143384, the *P-*value becomes insignificant ((effect allele A, EAF 0.64, OR [95% CI] 1.51 [1.33–1.70], *P* *=* 0.61), suggesting the association observed with rs143383 is not an independent signal. Since DDH is more prevalent in women, we also repeated the analysis using sex as a covariate and found no qualitative difference in the results (Supplementary Table 3).

### Replication

Independent association signals were taken forward to replication in three DDH cohorts of UK European ancestry, totalling 1129 cases and 4652 UKHLS controls. Following QC, one of the variants was excluded from further analysis due to low call rate. Five SNPs showed evidence for nominally significant (*P* < 0.05) association with the same direction of effect as the discovery cohort, compared with 1.35 under the null expectation (binomial test *P* = 0.01; Supplementary Table 4). rs143384 reached genome-wide significance in the replication dataset alone (effect allele A, EAF 0.61, OR [95% CI] 1.37 [1.24–1.51], *P* = 1.33 × 10^−10^). When the replication analysis was performed excluding the 66 NJR subjects that failed genome-wide genotyping QC, the findings were similar.

### Meta-analysis

**Fig. 3:**
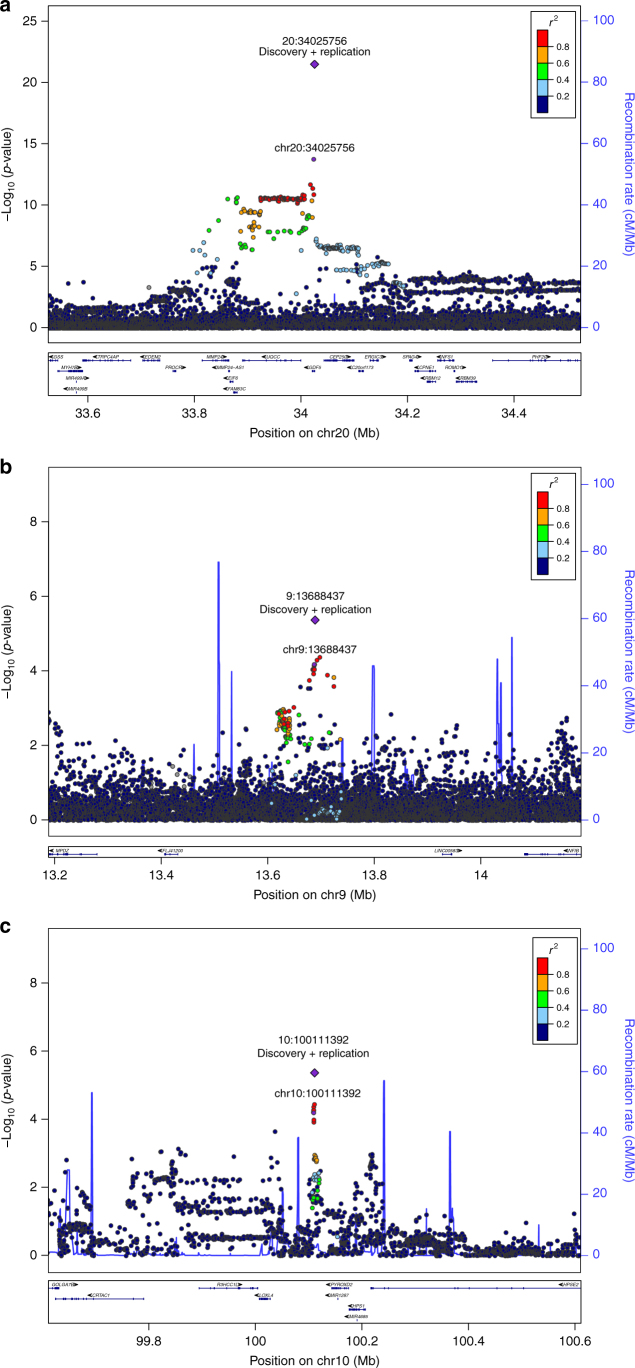
Regional association plots of the three replicating signals. Regional association plot of rs143384 (20:34025756) (**a**), rs4740554 (9:13688437) (**b**) and rs4919218 (10:100111392) (**c**). Each filled circle represents the *P*-value of analysed variants (as −log_10_ *P*-values) plotted against their physical position (NCBI Build 37). The *P*-value at the discovery stage and combined discovery and replication cohorts is represented by a purple circle and diamond, respectively. The other variants in the region are coloured depending on their degree of correlation (*r*^2^) with the index variant according to a scale from *r*^2^ = 0 (blue) to *r*^2^ = 1 (red). Gene location is annotated based on the UCSC genome browser

Twenty SNPs had the same direction of effect in both the discovery and replication analysis. Upon meta-analysis, three SNPs in the same region of chromosome 20 showed association with DDH at genome-wide significance: rs143384 in *GDF5* (effect allele A, OR [95% CI] 1.44 [1.34–1.56], *P* = 3.55 × 10^−22^; Fig. 3a) rs12479765 in *MMP24* (effect allele G, OR [95% CI] 1.33 [1.20–1.47], *P* = 3.18 × 10^−8^), and rs2050729 in *RMB39* (effect allele G, OR [95% CI] 1.41 [1.25–1.58], *P* = 1.15 × 10^−8^). Conditional analysis of rs12479765 and rs2050729 on the lead variant rs143384 attenuated their association with DDH. Although the linkage disequilibrium (LD) *r*^2^ correlations among these three variants are lower than 0.16 (Supplementary Table 5), the *P*-values after conditioning on rs143384 were *P* = 0.004 and *P* = 0.045 for rs2050729 and rs12479765, respectively, suggesting residual nominal independent association of these variants with DDH. Rs143384 explained 0.96% of DDH variance on the liability scale (*h*^2^*L*) and the sibling relative risk ratio (*λ*_s_) attributable to this variant was *λ*_s_ = 1.04 (Supplementary Fig. 2).

Two further independent signals showed evidence of replicating association with DDH, although they did not reach genome-wide significance at meta-analysis: rs4740554 (effect allele C, EAF 0.10, OR [95% CI] 1.30 [1.16–1.45], *P* = 4.44 × 10^−6^) and rs4919218 (effect allele C, EAF 0.31, OR [95% CI] 1.19 [1.10–1.28], *P* = 4.38 × 10^−6^; Fig. 3b, c; Table 1). rs4740554 and rs4919218 explained 0.36% and 0.24% of phenotypic variance (*λ*_s_ = 1.02 and 1.01), respectively (Supplementary Fig. 2).

**Table 1:** Replicating variants associated with DDH

SNP ID (chromosome: position)	Effect allele/other allele	Effect allele frequency (discovery stage)	Discovery odds ratio (95% CI)	Discovery *P-*value	Replication odds ratio (95% CI)	Replication *P-*value	Meta-analysis odds ratio (95% CI)	Meta-analysis *P*-value
rs143384 (20:34025756)	A/G	0.60	1.57 (1.40–1.77)	1.72 × 10^−^^14^	1.37 (1.24–1.51)	1.33 × 10^−^^10^	1.44 (1.34–1.56)	3.55 × 10^−^^22^
rs4740554 (9:13688437)	C/A	0.10	1.44 (1.22–1.71)	4.09 × 10^−05^	1.20 (1.03–1.39)	0.01	1.30 (1.16–1.45)	4.44 × 10^−^^6^
rs4919218 (10:100111392)	C/T	0.69	1.27 (1.13–1.43)	6.48 × 10^−05^	1.14 (1.03–1.25)	0.0089	1.19 (1.10–1.28)	4.38 × 10^−6^

### Fine-mapping

For the *GDF5* signal, the sum of probabilities of causality for five variants in the fine-mapped region was ≥0.95. Among these, both rs143384 and rs143383 have high epigenomic scores as they are located in the 5′ UTR of *GDF5*, have high nucleotide sequence conservation, and overlap DNase hypersensitivity and activating histone marks. Considering annotations and association *P*-values together, rs143384 had a >99% likelihood of being the causal variant, assuming only one variant as causal. For the other two replicating signals it was not possible to narrow down the location of the likely causal variants, as the chr10 and chr9 locus credible sets contain 483 and 618 variants, respectively.

### Gene-based analyses

We performed gene-based analysis using MAGMA v1.03 (ref. ^18^) to identify genes which contain multiple variants that contribute to DDH. Prior to gene analysis, MAGMA applies an internal SNP QC process. MAGMA uses all remaining SNPs in a gene to compute gene *P*-values while accounting for LD and after adjusting for the family-wise error rate (FWER). We found five genes that were significantly associated with DDH susceptibility: *GDF5*, *UQCC1*, *MMP24*, *RETSAT* and *PDRG1* (*P* = 9.24 × 10^−12^, *P* = 1.86 × 10^−10^, *P* = 3.18 × 10^−9^, *P* = 3.70 × 10^−8^ and *P* = 1.06 × 10^−7^, respectively). Following QC, 13, 18, 17, 20 and 6 SNPs were included in the analysis for *GDF5*, *UQCC1*, *MMP24*, *RETSAT* and *PDRG1*, respectively (Supplementary Fig. 3). To exclude association through *GDF5*, we repeated the analysis by removing *GDF5* and all genes remained significantly associated with DDH. We further looked up the functionality of the variants that were used in the analysis for each gene to better understand whether their effects are local to the gene itself, or may have more distant regulatory effects. Over 59% of these remaining variants in each gene analysed by MAGMA are assumed to have high (disruptive) impact in the protein (e.g. missense, stop codon) (Supplementary Fig. 3) and none of the significant genes share variants that are in high LD with each other across genes (Supplementary Table 6). Fourteen variants are expression quantitative trait loci (eQTL) that regulate various genes nearby (Supplementary Data 1).

### Genetic overlap with hip osteoarthritis

We calculated polygenic risk scores to detect shared genetic aetiology between DDH and hip osteoarthritis. We found no evidence that hip osteoarthritis polygenic risk score predicts DDH status. The best-fit polygenic risk score for hip osteoarthritis in the arcOGEN^19^ and in the UK Biobank (UKBB) International Classification of Diseases (ICD) 10 coded hip osteoarthritis datasets explained ~0.05% and 0.35% of variation in DDH susceptibility, respectively (FWER *P* > 0.05; Supplementary Fig. 4A, B). To further investigate the genetic overlap with hip osteoarthritis, we utilised LD score regression (LDSC)^20^ to estimate the genetic correlation between DDH and UKBB hip osteoarthritis datasets. DDH showed a nominally significant positive genetic correlation (rg) with hip osteoarthritis (rg = 0.5839, s.e. = 0.2068, *P* = 0.0047). However, this finding does not distinguish between shared genetic causes and a genetic causal relationship between the two traits. We also calculated the genetic correlations between DDH and 235 other traits and diseases using GWAS summary statistics and LD score regression implemented in the online software LD Hub^21^. After applying the false discovery rate (5% FDR) procedure to correct for multiple testing, none of the traits/diseases had a significant genetic correlation with DDH (Supplementary Data 2). We found a nominally significant genetic correlation with citrate (rg = 0.5934, s.e. = 0.2211, *P* = 0.0073), acetate (rg = −0.6778, s.e. = 0.2545, *P* = 0.0077), birth weight (rg = 0.2399, s.e. = 0.1104, *P* = 0.0298), infant head circumference (rg = 0.3932, s.e. = 0.1826, *P* = 0.0313) and child birth weight (rg = 0.3576, s.e. = 0.1815, *P* = 0.0488) which did not pass multiple-testing correction.

## Discussion

We used case-ascertainment by national clinical audit database search and postal recruitment to facilitate the largest genome-wide association scan for DDH to date. We find that the heritable component of DDH due to common autosomal variants is approximately 55%, consistent with the complex nature of the disease, and find evidence for genetic correlation but a lack of predictive power of osteoarthritis polygenic risk scores on DDH. We establish variation within *GDF5* on chromosome 20 as robustly associated with DDH susceptibility with rs143384 as the causal signal by fine-mapping, although *GDF5* variation makes only a small contribution to overall DDH heritability. Through gene-based analyses we identify *GDF5*, *UQCC1*, *MMP24*, *RETSAT* and *PDRG1* to be associated with DDH susceptibility.

The patients recruited in the discovery cohort were adults with a diagnosis of hip dysplasia ascertained via a national clinical audit database for hip replacement. We employed a stepwise filtration approach to mitigate misclassification bias in case ascertainment, using indication for surgery recorded in the NJR followed by self-reported questionnaires for phenotype confirmation. The discovery cohort studied here may reflect individuals with either untreated DDH or who have had treated disease eventually leading to the need for hip arthroplasty, resulting in potential selection bias. In mitigation against this, the female to male case ratio in our final NJR DDH cohort was similar to that expected in the general DDH population in the UK^2^, and contrasts with the sex ratio found in the population with idiopathic osteoarthritis of the hip^22^. The case filtration approach used was also validated with high concordance by examination of plain radiographic images acquired through the National Data Sharing Network. Ancestry screening by questionnaire in this population also resulted in only a small percentage of outliers requiring exclusion by genotyping. Finally, our replication cohorts mainly comprised independent populations of children recruited prospectively with a firm diagnosis of DDH, and in whom the EAF and odds ratio of association for the primary variant signal was almost identical as that found in the NJR discovery sample. DDH is a risk factor for degenerative disease at the hip^11,12,23^, and osteoarthritis susceptibility genes influence the association between hip morphology and osteoarthritis^15,24,25^. We therefore also examined whether we were simply detecting osteoarthritis-associated genetic loci in our DDH discovery cohort, as case identification required NJR registration for a hip replacement. Polygenic risk scores derived using independent hip osteoarthritis cohorts predicted only a small amount of the genetic variability in the DDH discovery population. However, using LDSC we found a 58% positive genetic correlation between the two diseases. The main difference between the two analyses is that PRS uses variants under a specific *P*-value threshold where LDSC uses genome-wide summary statistics. Although LDSC is a method to estimate the degree to which genetic risk factors are shared between two diseases or traits, it does not ascribe causation. Thus the observed correlation might be attributed either to shared genetic effects on both traits or because of causal pathways between traits. Moreover, it’s not clear if one variable is causal to the other or if causation is mediated by an independent factor. Future studies using Mendelian randomisation analyses may determine the causal relationships between DDH and hip osteoarthritis.

Previous studies examining the epidemiology of DDH within families using linkage approaches have demonstrated that heritable factors contribute between 50% and 85% of the total risk or liability of disease^3,26,27^, depending on population ancestry. Here, we used dense genome-wide genotyping data to estimate the heritable risk of DDH among unrelated individuals of UK European ancestry in the general English population. Our estimate of DDH heritability attributable to variation within common autosomal chromosomes was broadly similar to the estimates derived from linkage analyses, and confirms the complex nature of the disease^2^. Although DDH shows strong sexual dimorphism, the heritability estimate was almost identical (54.7 ± 5.8%) when the analysis was repeated using sex as a covariate. When the heritability analysis was stratified by chromosome, we found that the X-chromosome contributed a similar amount (adjusted for length) to overall DDH heritability as the autosomes (Supplementary Fig. 1), and we identified no individual chromosome X signals. We also conducted the discovery association analyses both with and without adjustment for sex (Supplementary Tables 2 and 3), and this made no qualitative difference to the results. Taken together, these data suggest that although DDH is a strongly sex-linked condition, this is not substantially due to variation in genomic DNA.

The gene *GDF5* encodes growth differentiation factor 5 (GDF5), belonging to the transforming growth factor beta superfamily^28^. GDF5 is required for normal bone and joint development by promoting cartilage condensation and increasing the size of the skeletal elements through proliferation within epiphyseal cartilage^29^. Two *GDF5* variants, rs143383 and rs143384, have previously shown suggestive association with DDH in unreplicated candidate gene studies in Asians and in Europeans^5,30^. Here we validate and extend these findings at the genome-wide level and with robust replication. These variants are in high linkage disequilibrium (*r*^2^ = 0.82, *D*′*′* = 0.99), and were both directly genotyped in this study. Our data implicate rs143384 as the lead variant, with the rs143383 association with DDH being two orders of magnitude weaker. Conditional analyses substantiate this observation.

We identify an extended region of 16 DDH-associated variants at genome-wide significance and in high LD extending across *GDF5* and *UQCC*, with rs143384 as the lead signal. Interrogation of the GTEx database indicates that rs143384 is an eQTL for multiple genes across various tissues (Supplementary Table 7)^31,32^. Recently, Chen et al.^33^ have shown that the *GDF5* locus contains many separate regulatory elements that control expression of the gene at different joint sites, and that these flanking regions are large. The same group have since described a novel enhancer region GROW1 in an extended downstream regulatory region of *GDF5*^34^. The lead variant in this 5′ enhancer is rs4911178 (Chr:Pos 20:33952620), a G > A substitution in which the variant allele A occurs at high frequency in Eurasian populations, and in which DDH and osteoarthritis are also common^34^. This variant was also directly typed in our discovery cohort, and identified in our extended association region on chromosome 20 (Supplementary Table 3). The effect allele G (OR 0.67, 95% CI 0.59–0.76, *P* = 2.98 × 10^−^^11^) was in strong LD (*r*^2^ = 0.8) with rs143384, consistent with the findings of Capellini et al.^34^. This annotated function provides novel opportunities for investigation of the role of GDF5 as a candidate target for DDH prevention.

Gene-based enrichment analysis can boost power to identify contributing loci by combining information across multiple SNPs co-localised at the gene level. We found significant associations between DDH and the *GDF5*, *UQCC1*, *MMP24*, *RETSAT* and *PDRG1* genes. The results of functional look up, LD calculations and after removing the *GDF5* locus from the analysis, tie the association to the above genes. However, these results refer only to the variants used in the gene-based analysis and further investigation through functional experiments is warranted to asses if these signals are truly independent. *UQCC1* gene encodes a trans-membrane protein ubiquinol-cytochrome-*c* reductase complex chaperone, which is structurally similar to the mouse basic fibroblast growth factor repressed ZIC-binding protein. *UQCC* is expressed in differentiating chondrocytes and regulates growth control in mouse^35,36^. In humans, polymorphisms in this gene are associated with DDH^10^, bone size^37^, height^38^ and hip axis length^39^. *MMP24* encodes a member of the peptidase M10 family of matrix metalloproteinases (MMPs) which are involved in the breakdown of extracellular matrix in normal physiological processes, such as embryonic development and tissue remodelling^40^. Polymorphisms within *MMP24* have also been associated with height variation in childhood^41,42^. *RETSAT* codes for retinol saturase, an enzyme centrally involved in the metabolism of vitamin A^43^. Retinoic acid signalling is essential for normal limb bud development, including bone and cartilage formation^44^. The *RETSAT* pathways provides a further candidate therapeutic target for the prevention of DDH.

In conclusion, this largest study of DDH to date provides a comprehensive picture of its complex genetic architecture, the contribution of common variants to its heritability, and establishes the first robust DDH genetic locus to our knowledge. We demonstrate proof of principle for the utility of national clinical audit-based case ascertainment and recruitment for genetics and genomics studies using a strategy that is readily transferrable to other complex diseases in which large-scale datasets are held.

## Methods

### Study oversight

The study was approved by the National Research Ethics Service in England (NRES 12/YH/0390, 30 October 2012). Initial contact was made by the NJR data controller and all subjects provided written informed consent prior to participation.

### Study populations

*National Joint Registry (NJR) cohort*: The NJR hip dysplasia base population comprised 5411 adult individuals (4095 females) living in England who had undergone a hip replacement recorded in the NJR by the operating surgeon as being for the indication of hip dysplasia and who had given written permission for re-contact for research purposes. All were invited to participate in the postal screening phase of the study between January 2013 and April 2014 (Fig. 1, recruitment flowchart). Screening for UK European descent was made using self-declared ancestry as: White European, Black African, Black Caribbean, Asian, Arabic, Chinese/Oriental, Mixed, or Other; and supplemented by country of birth, mother’s country of birth, and father’s country of birth. A further check for ethnicity outliers was made at genotyping, where DNA samples proceeded to analysis. DDH history was confirmed by self-completed questionnaire (Supplementary Table 8). Inclusion required a positive response to both question 1 and 2. Subjects responding positively to any items within questions 6, 7 or 10 were excluded from further participation. One thousand and ninety-one individuals confirming both UK European ancestry and idiopathic DDH diagnosed in childhood were invited to return by post a saliva sample for DNA extraction and genotyping. Nine hundred and seven subjects (mean age 51.6 ± 12.4 years; 803 (88%) females) returned a saliva sample for DNA processing. The sex distribution of the subjects returning a sample was similar to that of the DDH population in the UK^2^. In 231 subjects (mean age 51.6 ± 12.4 years; 211 (91%) females) operated in hospitals from which >10 cases were recruited and independent radiographic validation of the DDH phenotype was made following retrieval of the pelvic radiograph preceding hip replacement using the National Data Sharing Network (http://www.image-exchange.co.uk/). The images were reviewed independently by two experienced orthopaedic surgeons for the presence and grade of DDH using the Hartofilakidis^45^ classification. A third observer made the final judgement if there was disagreement. This analysis confirmed DDH in 224 (97%) of subjects. Eighty had radiographic evidence of a previous corrective pelvic and/or femoral osteotomy that precluded DDH grade classification beyond confirmation of the DDH phenotype. Of the remaining 151 subjects, 77 showed Hartofilakidis grade A dysplasia (femoral head contained within the original acetabulum despite the degree of subluxation), 48 were of grade B (low dislocation, femoral head articulates with a false acetabulum that partially covers the true acetabulum to a varying degree), and 31 were of grade C (high dislocation, femoral head is completely out of the true acetabulum and migrated superiorly and posteriorly to a varying degree). Seven subjects were excluded from further analysis, and all had a radiographic phenotype of idiopathic osteoarthritis. This distribution of disease grades was consistent with expectation of an adult patient cohort with a history of childhood DDH^2,46,47^. The mean age of the 770 subjects (693, 90% females) participating in the discovery GWAS was 50 ± 12 years.

*DDH case control cohort:* This population comprised 838 children (725 female) mean age 7 ± 5 years recruited by face-to-face contact at 1 of 18 participating hospitals (see related manuscript file) and undergoing hospital treatment for DDH, or currently under follow-up for hospital-treated DDH. These subjects had been previously diagnosed with DDH by patient history and clinical examination in conjunction with radiography or ultrasonography. Subjects with borderline forms of DDH, such as a brief episode of harness treatment for mild instability, were excluded. None of the participants in the DDH case control cohort have undergone hip replacement and are therefore independent of the participants in the NJR cohort.

*Royal National Orthopaedic Hospital (RNOH) cohort:* The subjects within this cohort comprised 225 adults (220 female), mean age 32 ± 16 years participating in the RNOH DDH biobank, with a clinician-confirmed childhood history of idiopathic DDH by patient history and clinical examination in conjunction with radiography or ultrasonography, and undergoing osteotomy for residual dysplasia and/or degenerative changes. Subject level identification checks between the participants in the RNOH versus NJR adult recruitment cohorts was used to exclude any subject overlap between the cohorts.

The replication cohort comprised the 838 children from the UK DDH case control consortium (DDH CCC) and the 225 adults with DDH recruited in the RNOH DDH biobank. In addition, 66 cases (59 female) from the NJR discovery cohort that were of insufficient DNA yield for genome-wide genotyping were also included in the replication cohort. The mean age and sex distribution of the 66 NJR subjects participating in the replication cohort was similar (age 50 ± 14 years, 59 (89%) females) to that participating in the NJR discovery cohort. Subject overlap between the discovery and replication populations, and between the replication cohorts was excluded using individual patient identifiers and by cross-referencing to the NJR dataset. Case duplication was excluded in the discovery cohort at both subject ID and genotype quality control stages.

The control population comprised 8016 participants from the UKHLS. A sample of 3364 UKHLS controls were included in the discovery stage and an independent sample of 4652 UKHLS controls was used in the replication stage at a case:control ratio of ~1:4. The proportion of female controls in the discovery stage was selected to reflect the distribution in cases. All replication and control participants were of UK European ancestry.

*arcOGEN cohort:* The arcOGEN dataset comprises 7410 unrelated men and women with osteoarthritis and 11008 unrelated controls from the UK^19^. The osteoarthritis hip strata comprises 3266 cases with symptomatic hip osteoarthritis and a radiographic KL score ≥2. The arcOGEN study used two different types of controls: population-based, unrelated UK controls which came from five distinct sources: the 1958 Birth Cohort (58BC) and the UK Blood Donor Service (UKBS) from the Wellcome Trust Case Control Consortium 2 (WTCCC2) study, the 1958 Birth Cohort from the Type 1 Diabetes Genetics Consortium (T1DGC) study, the Avon Longitudinal Study of Parents and Children (ALSPAC) and the People of the British Isles (PoBI) study (ST1); and unrelated, osteoarthritis-free controls (females only) from the TwinsUK cohort which comprises twins ascertained to study the heritability and genetics of age-related diseases^48^. These unselected twins were recruited from the general population through national media campaigns in the UK and shown to be comparable to age-matched population singletons in terms of disease-related and lifestyle characteristics^49^.

*UK Biobank cohort*: The UK Biobank is a prospective cohort of 500,000 men and women aged between 40 and 69 years at recruitment^50^. Hospital episode statistics were used to define case status for osteoarthritis in the UKBB sample. Inclusion and exclusion criteria were based on the International Statistical Classification of Diseases and Related Health Problems (ICD) 9 or ICD-10 codes. In total, 2396 cases were defined with a code for hip osteoarthritis, and no inflammatory arthritis syndromes or other musculoskeletal disorders. Age-matched controls were selected on the condition that they did not have any osteoarthritis-related ICD-9 or ICD-10 codes, or self-reported musculoskeletal disorders or symptoms.

### Genotyping, quality control, and association analyses

DNA from 834 NJR DDH cases was genotyped using the Illumina HumanCoreExome-24 BeadChip (Illumina, San Diego, USA). Genotypes were called using Illumina Genome Studio Gencall. The UKHLS were genotyped on the Illumina HumanCoreExome-12v1-0_A chip and had previously been called using the same algorithm. After removing samples and variants with call rate <90%, samples underwent standard quality-control procedures, with exclusion criteria as follows: (i) call rate <98%, (ii) gender discrepancy, (iii) excess heterozygosity, (iv) duplicates and/or related, (v) ethnicity outliers, (vi) Fluidigm concordance (this identity check looks at sample concordance between Illumina and Fluidigm genotypes) (Supplementary Table 9) and variants with (i) call rate <98%, (ii) Hardy–Weinberg Equilibrium (HWE) *P* < 1 × 10^−^^4^, (iii) cluster separation score <0.4, (iv) minor allele frequency (MAF) <0.01, (v) minor allele count (MAC) <4 in cases and controls separately.

Data were pruned for linkage disequilibrium using the clumping function in PLINK^51^. Parameters used: (a) significance threshold for index single single-nucleotide polymorphism (SNP): 1e-4, (b) LD threshold for clumping: 0.20, and (c) physical distance threshold for clumping: 500 kb.

Following quality-control exclusions, the discovery cohort comprised 770 (693 females) DDH cases and 3364 (3048 females) controls. Power was calculated using Quanto v1.2.4^52^, assuming a population mean of 0 and a standard deviation (SD) of 1. We calculated power (at the genome-wide significance threshold *P* = 5 × 10^-8^) to detect an effect size of 1 SD by fixing the sample size to the post-QC discovery sample, which gave >80% power to detect common variants with moderate effect size (Supplementary Fig. 5). Following QC checks at both the sample and SNP level, 256,867 overlapping variants were tested for association with DDH using SNPTEST v2^53^ under an additive model and using a maximum likelihood ratio test. Association testing on the X chromosome assumes a model of full X inactivation where pseudo-autosomal regions on the X were treated like autosomes. The Y chromosome was not included in the analysis. Cluster plots of all prioritised variants were examined in cases and controls separately to minimise the possibility of spurious association due to genotyping error. Independent variants with *P* < 1 × 10^−^^4^ in the discovery set were selected for replication using the clumping function in PLINK.

*De novo* genotyping (prioritised variants genotyped in independent cohorts) in the 1129 replication cases was conducted using the iPLEX® Assay and the MassARRAY® System (Agena Bioscience, Inc). Variants with poor Agena design metrics were replaced with highly correlated proxies. Sample exclusions were based on sex inconsistencies and a sample call rate <80%. Variants with a call rate <90% and exact HWE *P* < 1 × 10^−6^ were also excluded. Case control association analysis in the replication cohort of 1129 (1004 females) cases and 4652 (2527 females) controls was performed under an additive genetic model using SNPTEST v2. When the replication analysis was performed excluding the 66 NJR subjects who failed genome-wide genotyping QC, the findings were similar (Supplementary Table 10). Finally, a fixed-effects meta-analysis across the discovery and replication datasets was conducted using GWAMA^54^, comprising a total of 1899 cases and 8016 controls. Genome-wide significance was defined as *P* < 5.0 × 10^−^^8^. Conditional single-variant association analyses were performed in SNPTEST v2 and were used to identify statistical independence. SNPTEST performs conditioning of a signal by adding any potentially dependent SNP as a covariate in the analysis. It then tests for a genetic effect over and above the phenotypic variability that is explained by the covariate(s). A variant was considered independent of the index SNP if the pre- and post-conditioning *P*-value difference was lower than two orders of magnitude. We repeated the analysis by adjusting for the first 10 principal components to guard against population stratification. We observed no qualitative difference and report the results of the unadjusted analysis. Since DDH prevalence varies widely between populations, we applied multidimensional scaling analyses to characterise our samples (cases and controls) in the context of broader global diversity and further ensure the homogeneity of the tested cohort. As shown in Supplementary Fig. 6 our cohort formed a well-defined cluster with GBR population of 1000 Genomes Project.

### Estimation of heritability

We applied the genome-wide complex trait analysis (GCTA) approach to estimate the proportion of phenotypic variance in DDH susceptibility that is explained by genome-wide autosomal SNPs^17^, assuming a DDH prevalence of 3.6 per 100 live births in the UK^2^, and using an MAF cut-off of 0.01. GCTA uses a genetic relationship matrix (GRM) of pairs of samples as input for the restricted maximum likelihood (REML) analysis to estimate narrow sense heritability (*h*^2^). GCTA also converts *h*^2^ to liability-scale heritability by applying the transformation described by Lee et al.^55^, to make the estimates comparable with those for quantitative traits. An alternative approach based on phenotype correlation–genotype correlation (PCGC)^56^ regression has been proposed to avoid biases in REML estimation of heritability in the context of ascertained case–control studies. Here, we applied both GCTA and PCGC methodologies in order to validate our results and ensure that the heritability estimates did not suffer from case–control ascertainment bias. The sibling relative risk ratio and liability-scale variance explained were calculated according to the methods described by Morris et al.^57^. To investigate if the DDH strongly sex-linked condition is due to variation in genomic DNA, we further partitioned the genetic variance onto individual chromosomes and then compared them to heritability estimates of chromosome X. In this analysis, GCTA uses a joint analysis (all the chromosomes are fitted in one model) to protect against inter-chromosomal correlations and report the estimates of variance explained by different chromosomes independently of each other. GCTA contains built-in options for GRM and heritability estimation for the X chromosome and assumes a model of full X inactivation^58^.

### Fine-mapping

In order to fine-map the replicating association signals, we used association statistics from imputed genotypes in determining the credible set, since an assumption of fine-mapping is that the causal variant is among the variants tested. All variants within 500 kb of the three replicating lead SNPs were pre-phased in SHAPEITv2^59^ and the resulting haplotypes were imputed with IMPUTE2^60^ using 1000 Genomes Project^61^ combined with UK10K Project^62^ data as the reference panel. Post-imputation quality control (QC) criteria for SNP exclusion were HWE *P-*value <1.0 × 10^−4^ and imputation info score <0.4. We then applied a method that assigns a relative “probability of regulatory function” (PRF) score among candidate causal variants, reweighting association statistics based on epigenomic annotations, and delineating the 95% probability set of likely causal variants. We collected a set of 70 genomic and epigenomic annotations, primarily Gencode gene annotations (v19), FANTOM transcription start sites and enhancers^63,64^, and imputed Roadmap Epigenomics histone marks, DNase hypersensitivity, and ChromHMM genome segmentations for the lymphoblastoid cell line epigenome (GM12878)^65,66^. We used the fgwas software^67^ to train a Bayesian hierarchical model to compute enrichment of eQTLs in these annotations based on summary statistics from the Geuvadis RNA sequencing project^68^. We used forward stepwise selection followed by cross-validation to arrive at a combined model with 37 annotations and their associated enrichments. The respective annotations from 119 Roadmap epigenomes were used to compute PRF scores for each variant at the new loci in each of the 119 epigenomes. At each locus we defined the credible set of causal variants using the WTCCC method with approximate Bayes factors (BF)^69^, where SNP *k*’s posterior probability of being causal is $BFk/\mathop {\sum }\limits_j BFj$. Beginning with the most associated SNP, SNPs are sequentially added to the set until the combined probability is ≥95%. We then selected the top four epigenomes based on the maximum regulatory score among variants in the 95% credible set, and examined the regulatory annotations for variants in the credible set.

### Gene-based analyses

In order to quantify the degree of association each gene has with the phenotype and understand the potential functional effects of the identified variants, we used the GWAS results to perform gene-based enrichment analyses in MAGMA v1.03^18^ to identify the joint association of all markers in a given gene with the phenotype. We used all 19,427 protein-coding genes from the NCBI 37.3 gene definitions and each gene was assigned the SNPs located between the gene’s start and stop sites. We used all variants that passed QC and after SNP annotation there were 17,516 genes that were covered by at least one SNP. Gene-based analysis was then carried out based on a multiple principal components regression model that accounts for linkage disequilibrium between SNPs, using an F-test to compute the gene-based *P*-value (self-contained *P-*value). *P*-values generated by MAGMA are not corrected for multiple testing. Since Bonferroni correction is conservative when variables (genes) are correlated, MAGMA provides a built-in FWER method which uses a permutation procedure to obtain *P*-values corrected for multiple testing. In all, 10,000 permutations were used in the analysis and the significance threshold was set at *α* = 0.05 (FWER threshold = 2.29^−07^).

### Genetic overlap with hip osteoarthritis

Polygenic risks scores were created for all genotyped participants to test for shared genetic aetiology with idiopathic hip osteoarthritis. Polygenic risk scores were calculated as the summation of an individual’s genotype across many genetic loci using PRSice^70^, and weighted by the effect size estimated from two independent UK-based cohorts: arcOGEN hip osteoarthritis cases versus controls and UK Biobank hip osteoarthritis cases versus controls, as outlined in the Study Populations section above. Age-matched controls were selected on the condition that they did not have a hospital diagnosed (ICD-9 or ICD-10) or self-reported musculoskeletal disorders or symptoms. Before creating the scores, the analysis was restricted to autosomal SNPs and clumping was used to obtain SNPs in linkage equilibrium with an *r*^2^ < 0.1 within a 250-bp window. Several polygenic scores were created containing SNPs according to the significance of their association with the hip osteoarthritis. Logistic regression models were used to test the associations between the polygenic profiles of hip osteoarthritis and DDH and the first two PCs were used to adjust for population structure. Nagelkerke’s pseudo-*R*^2^ calculation as a measure of the variance explained and the most predictive threshold are shown in Supplementary Fig. 4A, B. The genetic correlation between DDH and hip osteoarthritis was estimated using LD score regression by Bulik-Sullivan et al.^20^. This method exploits the LD structure of SNPs across the genome and uses the cross-products of summary test statistics provided from two GWASs at each SNP and then regresses the cross-product on the LD score to calculate the genetic correlations between traits/diseases. To test whether DDH has a shared genetic aetiology with 235 other diseases, we used LD score regression as implemented in the online software LDHub^21^ (http://ldsc.broadinstitute.org/). For both analyses we used summary statistics of the imputed DDH dataset. Genotype imputation was performed on the Michigan Imputation Server (http://www.haplotype-reference-consortium.org/) using the updated Haplotype Reference Consortium^71^. The combined UK10K/1000 Genomes Project haplotype reference panel was used to impute UKBB dataset centrally (http://www.ukbiobank.ac.uk/wp-content/uploads/2014/04/imputation_documentation_May2015.pdf).

### Data availability

Genome-wide genotype data of the DDH cases and UKHLS controls have been deposited to the European Genome-Phenome Archive (www.ebi.ac.uk/ega/home, DDH accession numbers: EGAD00010000766 and EGAS00001000916; UKHLS accession numbers: EGAD00010000891 and EGAS00001001232) and of the DDH NJR cases to the NJR data archive (www.njrcentre.org.uk).

## Electronic supplementary material

Supplementary Information

Description of Additional Supplementary Files

Supplementary Data 1

Supplementary Data 2
