# Supplementary material for: Genome-wide association study of developmental dysplasia of the hip identifies an association with GDF5
Source: Commun Biol. 2018 May 31;1:56. doi: 10.1038/s42003-018-0052-4 (PMC6123669; doi:10.1038/s42003-018-0052-4)
Supplement: Supplementary file 1 — Supplementary Information [file 42003_2018_52_MOESM1_ESM.pdf]

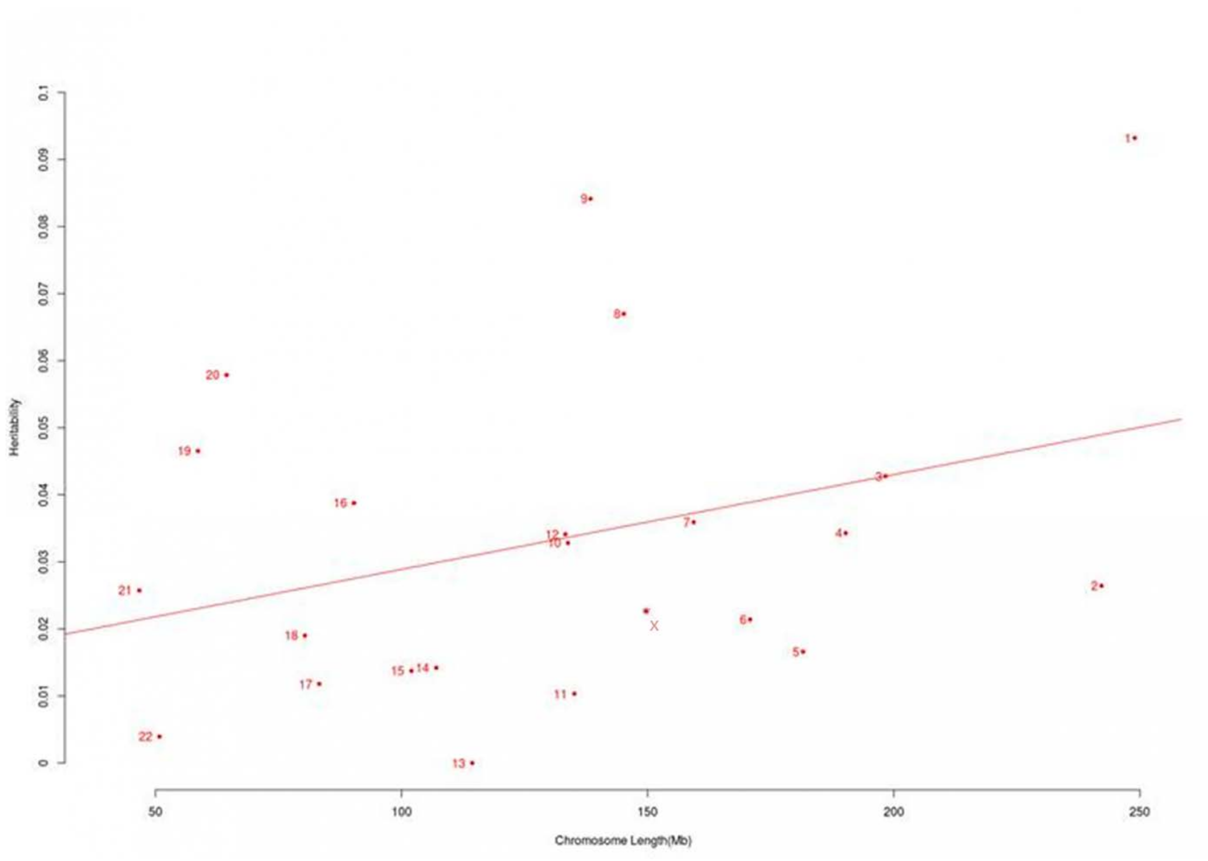

Supplementary Figure 1. Estimate of chromosomal heritability of all 22 autosomes plus X chromosome as a function of the genetic length of the chromosome.

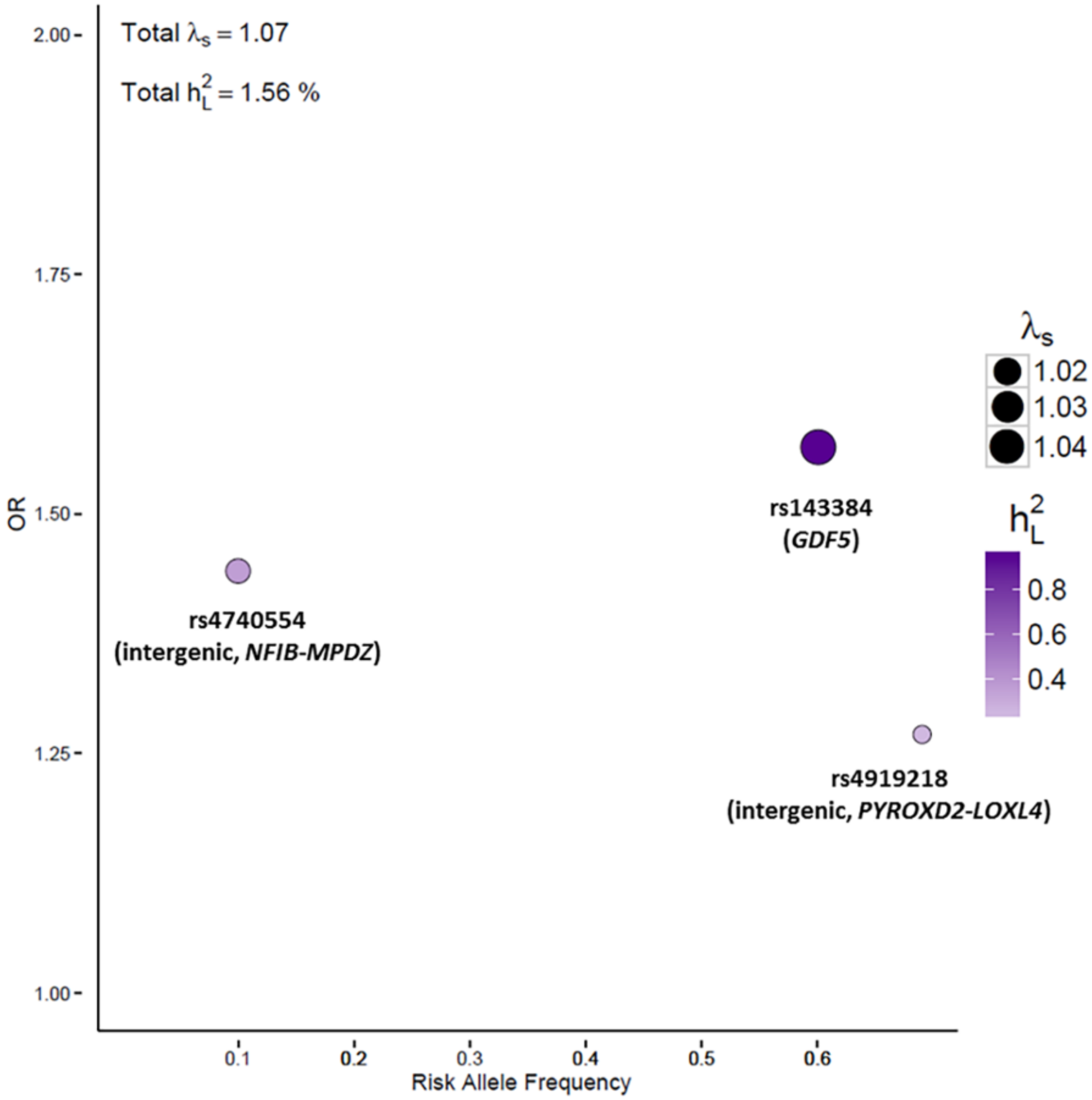

Supplementary Figure 2. Effect sizes (y axis) and risk allele frequencies (RAF, x axis) of rs143384, rs4740554 and rs4919218. Circle size is proportional to the sibling relative risk ratio (λ_s). Circle colour corresponds to the percentage of variance explained on the liability scale (h^2_L).

GENES	P value	NSNPS[a]	Consequence type/Count
GDF5	$9.24x10^{-12}$	13	5 prime UTR variant/2; missense variant/11
UQCC1	$1.86x10^{-10}$	18	Splice donor variant/1; splice acceptor variant/1; stop gained/2; missense variant /8; splice region variant/1; intron variant/5
MMP24	$3.18x10^{-09}$	17	Missense variant/10; splice region variant/1; synonymous variant/1; 3 prime UTR variant/1; intron variant/4
RETSAT	$3.70x10^{-08}$	20	Splice acceptor variant/1; stop gained/2; missense variant/17
PDRG1	$1.06x10^{-07}$	6	Splice acceptor variant/1; missense variant/5

[a] the number of SNPs annotated to that gene that were included in the analysis

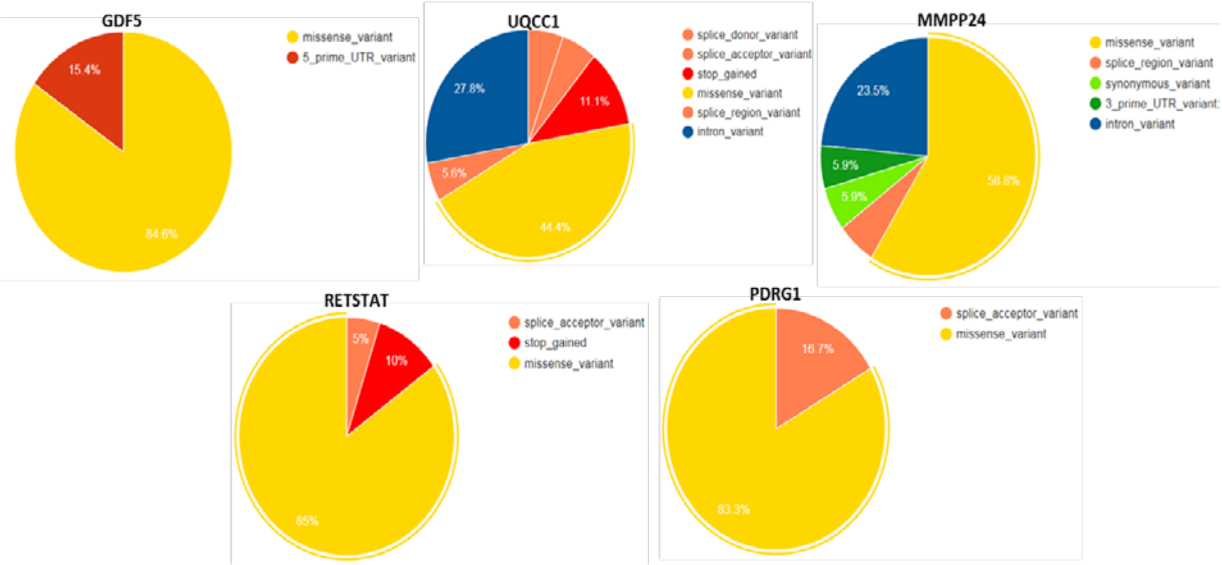

Supplementary Figure 3. Genome-wide significant autosomal gene-based hits (FWER correction of $\alpha < 2.29^{-07}$) in the MAGMA gene-based analysis for DDH. NSNPS column represents the number of SNPs that were finally analysed for each gene after MAGMA's internal QC.

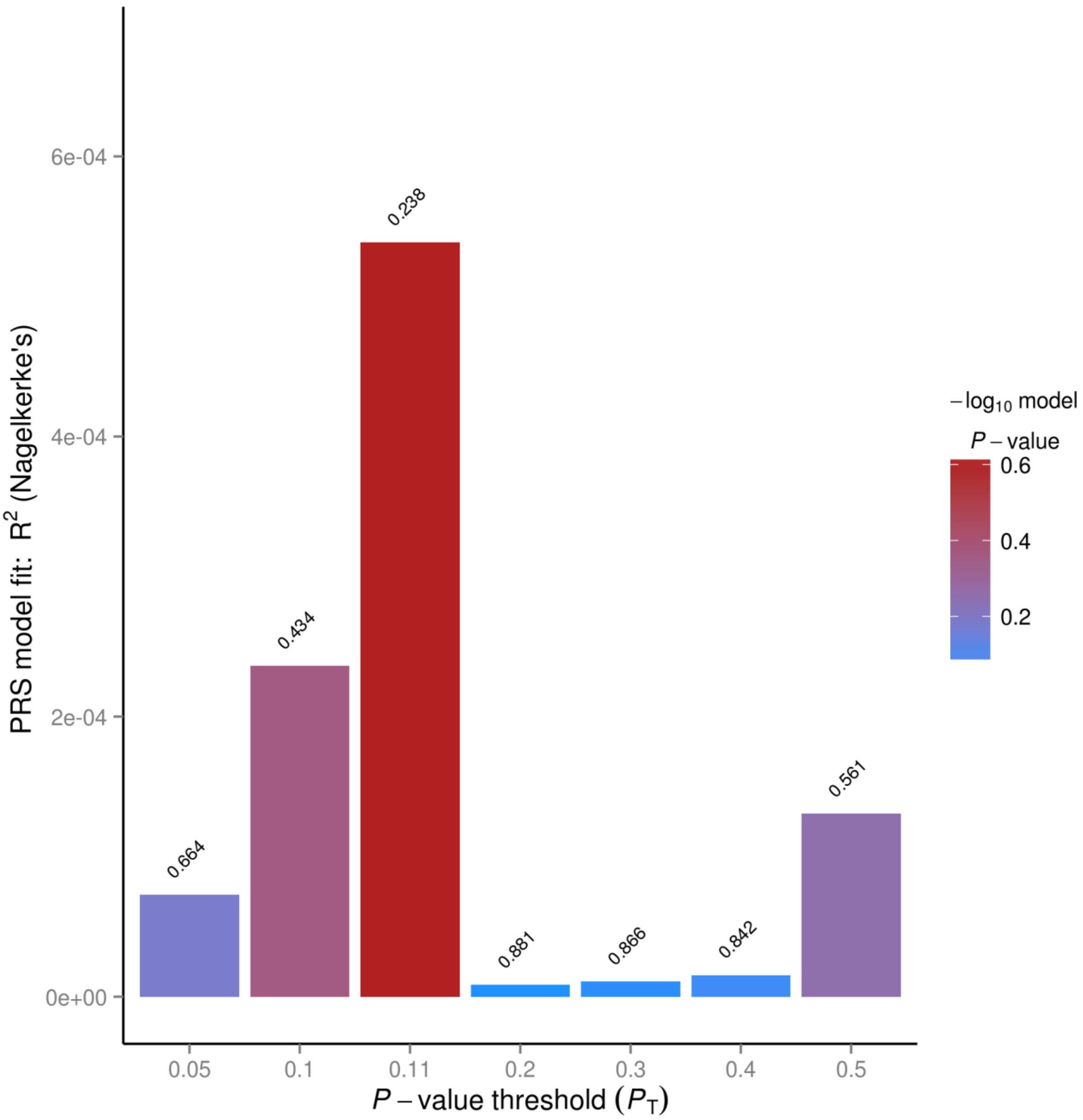

PRS model fit: R2 (Nagelkerke's)
6e-04
4e-04
2e-04
0e+00
0.664
0.434
0.238
0.881
0.866
0.842
0.561
0.05
0.1
0.11
0.2
0.3
0.4
0.5
P − value threshold (PT)
− log10 model
P − value
0.6
0.4
0.2

A

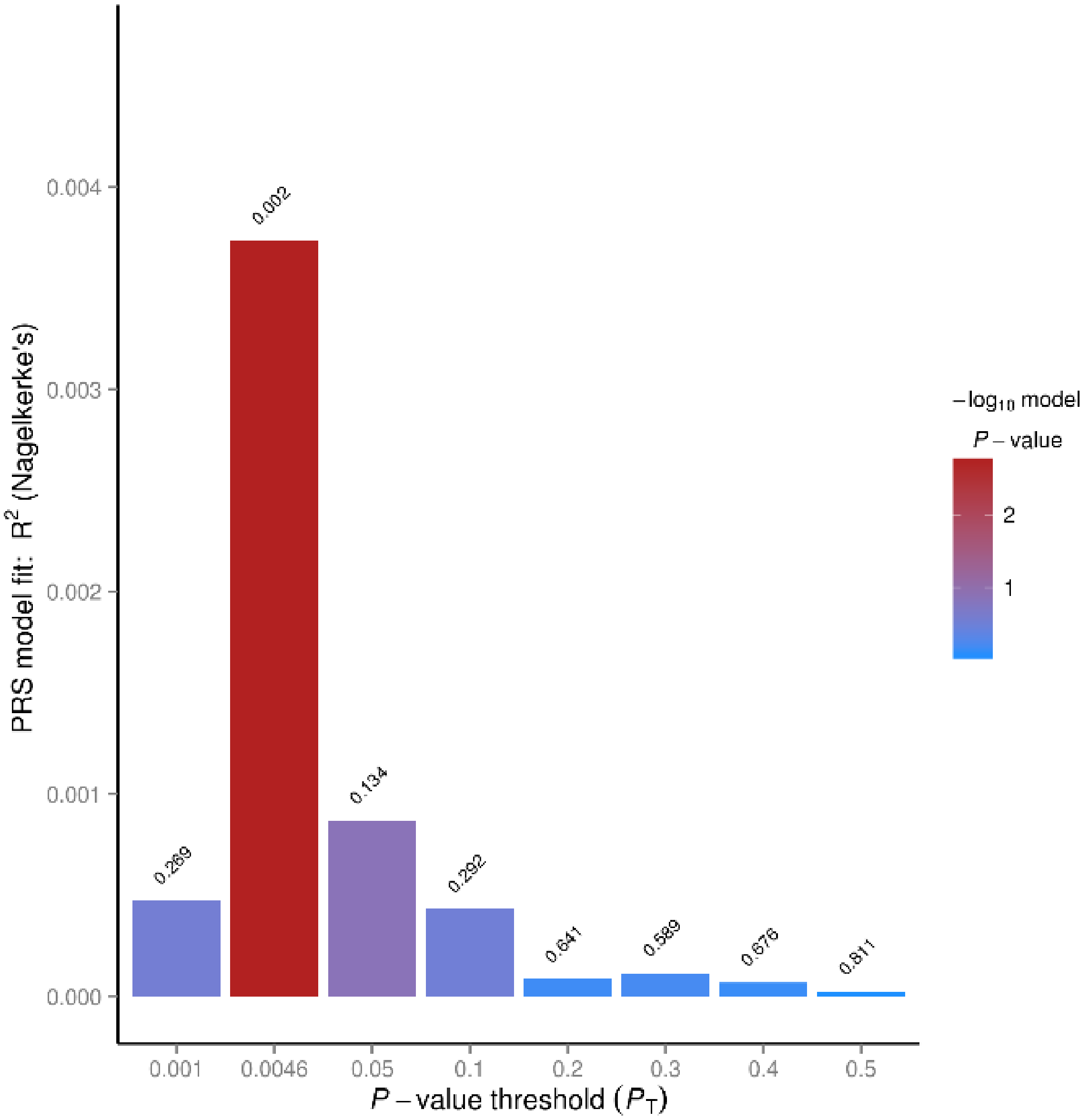

B

Supplementary Figure 4. Bar plot from PRSice high-resolution run in various *P*-value thresholds. The red bar shows the most predictive threshold for hip-OA predicting DDH. Panel A = comparison against arcOGEN dataset, Panel B = comparison against UK Biobank ICD-10 hip OA dataset.

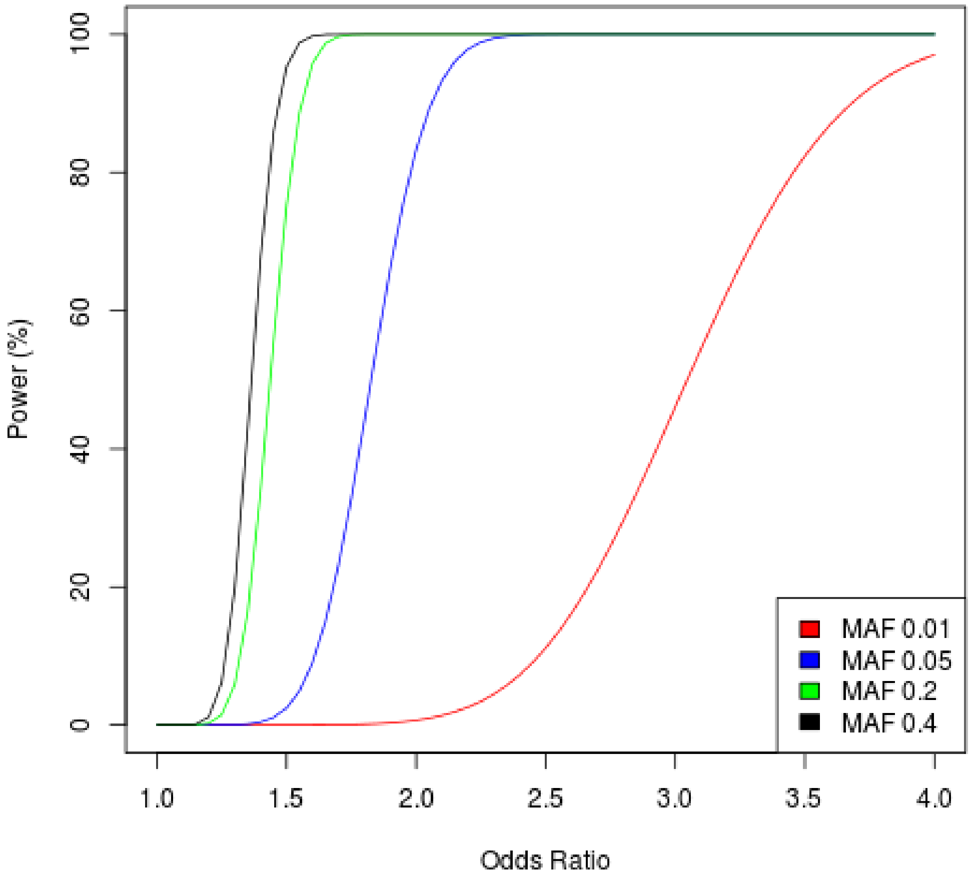

Supplementary Figure 5. Power calculations for the DDH discovery GWAS (770 cases and 3364 controls) at varying minor allele frequency and odds ratios.

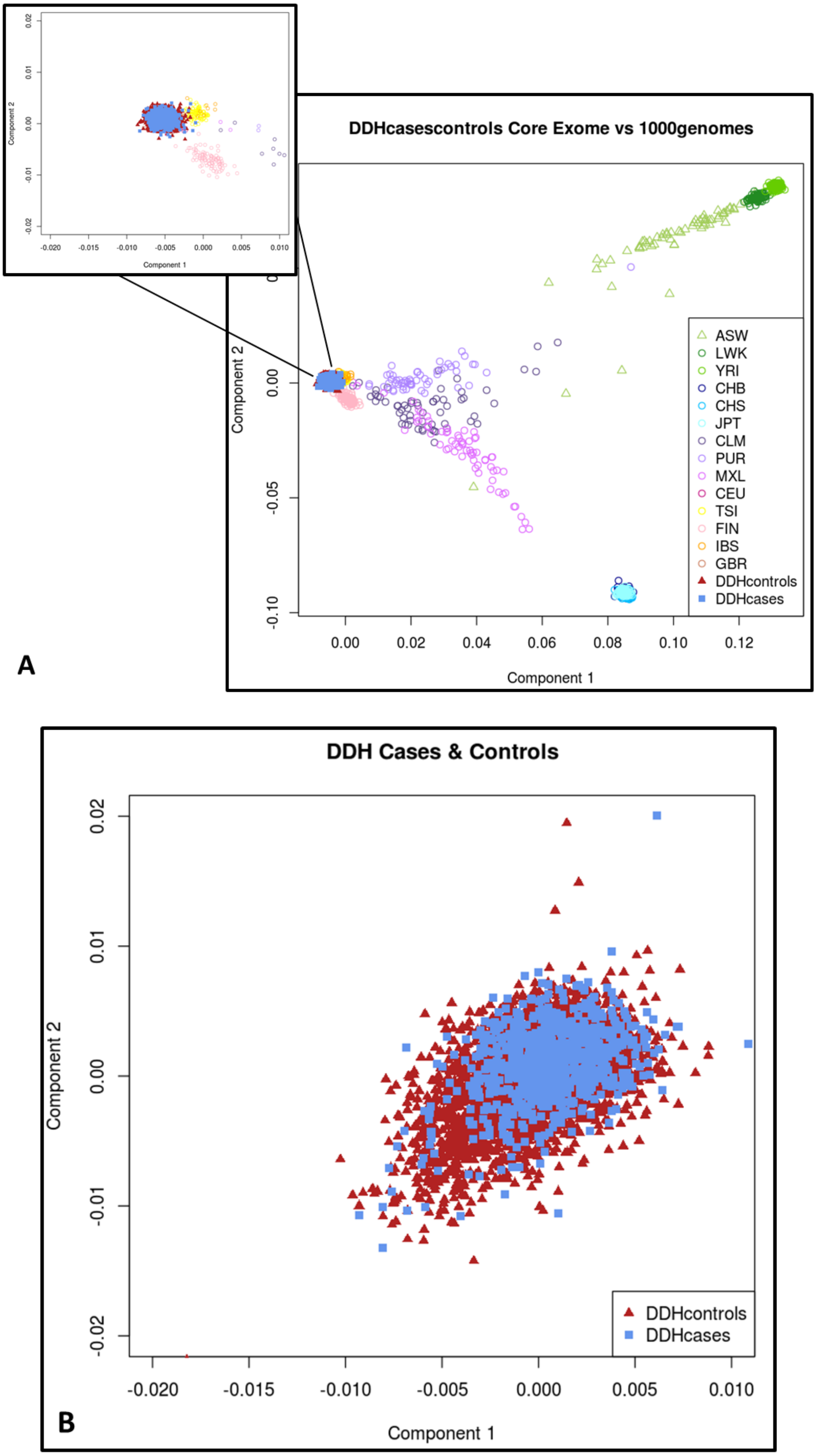
A
DDHcasescontrols Core Exome vs 1000genomes
Component 1
Component 2
0.00
0.02
0.04
0.06
0.08
0.10
0.12
0.00
-0.05
-0.10
ASW
LWK
YRI
CHB
CHS
JPT
CLM
PUR
MXL
CEU
TSI
FIN
IBS
GBR
DDHcontrols
DDHcases
B
DDH Cases & Controls
Component 1
Component 2
-0.020
-0.015
-0.010
-0.005
0.000
0.005
0.010
0.02
0.01
0.00
-0.01
-0.02
DDHcontrols
DDHcases

Supplementary Figure 6. A) Multidimensional scaling analysis of DDH cases and controls combined with populations from the 1000 Genomes Project. The firebrick solid triangles and the cornflower blue squares depict controls and cases from the DDH cohort, respectively. Individuals of 1000 Genomes Project are depicted by the differently colored hollow triangles/circles with each colour corresponding to the 14 worldwide populations. Abbreviations: ASW, Americans of African ancestry in southwestern USA; LWK, Luhya in Webuye, Kenya; YRI, Yoruba in Ibadan, Nigeria; CHB, Han Chinese in Beijing, China; CHS, Southern Han Chinese; JPT, Japanese in Tokyo, Japan; CLM, Colombians from Medellin, Colombia; PUR, Puerto Ricans from Puerto Rico; MXL, Mexican ancestry from Los Angeles, USA; CEU, Utah residents with Northern and Western European ancestry; TSI, Toscani in Italy; FIN, Finnish in Finland; IBS, Iberian population in Spain; and GBR, British in England and Scotland. B) Multidimensional scaling analysis of DDH cases and controls. The firebrick solid triangles and the cornflower blue squares depict controls and cases from the DDH cohort, respectively.

Supplementary Table 1. Heritability estimated by Genetic Complex Trait Analysis (GCTA) and Phenotype Correlation Genotype Correlation (PCGC) software.

**Software**	**Liability-scale heritability**[a] **(K=0.0036 (3.6 cases per 1000 births))**
GCTA	0.55 (±0.06)
PCGC	0.53 (± NA)

[a]estimate taking into account disease prevalence (K) and case-control ascertainment

Supplementary Table 2. Summary of loci associated with DDH in the discovery GWAS.

Chr: Position	EA	NEA	EAF Cases	EAF Controls	OR [95% CI]	*P* value
20:34025756[a]	A	G	0.69	0.58	1.57 [1.30-1.77]	1.72E-14
20:34025983	A	G	0.71	0.62	1.51 [1.33-1.70]	1.29E-11
20:34001058	T	C	0.71	0.62	1.50 [1.33-1.69]	1.31E-11
20:33864484	G	A	0.33	0.43	0.67 [0.60-0.76]	2.09E-11
20:33952620	G	A	0.29	0.38	0.67 [0.59-0.76]	2.73E-11
20:33975181	C	A	0.29	0.38	0.67 [0.59-0.76]	2.73E-11
20:33971914	T	C	0.71	0.62	1.49 [1.32-1.68]	2.90E-11
20:34001250	G	A	0.29	0.38	0.67 [0.60-0.76]	3.69E-11
20:33894463	G	A	0.30	0.38	0.68 [0.61-0.77]	2.37E-10
20:33907161	T	C	0.70	0.62	1.46 [1.30-1.65]	2.61E-10
20:33914208	G	A	0.30	0.38	0.69 [0.61-0.77]	3.58E-10
20:34048161	T	C	0.18	0.24	0.70 [0.60-0.80]	2.20E-07
20:34059675	G	A	0.18	0.24	0.70 [0.61-0.80]	2.70E-07
20:34097353	T	C	0.82	0.76	1.43 [1.24-1.65]	2.72E-07
20:33909784	T	C	0.24	0.30	0.72 [0.63-0.82]	3.89E-07
20:33799280	T	G	0.64	0.57	1.33 [1.19-1.50]	6.39E-07
14:96035911[a]	T	C	0.08	0.05	1.75 [1.41-2.19]	1.34E-06
12:1995403[a]	T	C	0.14	0.10	1.50 [1.28-1.77]	1.73E-06
1:35208698[a]	T	C	0.43	0.36	1.31 [1.17-1.46]	3.10E-06
20:34143092	G	A	0.18	0.23	0.73 [0.63-0.84]	6.01E-06
14:96085342	T	C	0.91	0.94	0.62 [0.51-0.76]	7.73E-06
20:33825378[a]	G	A	0.87	0.82	1.42 [1.21-1.66]	1.09E-05
14:88651006[a]	T	C	0.64	0.70	0.77 [0.69-0.87]	1.10E-05
1:85716807[a]	G	A	0.84	0.8	1.38 [1.19-1.61]	1.15E-05
21:47614553[a]	G	A	0.97	0.95	1.96 [1.41-2.72]	1.20E-05
16:78430875[a]	T	G	0.91	0.88	1.51 [1.24-1.83]	1.45E-05
20:34075998	T	C	0.87	0.82	1.41 [1.20-1.65]	1.70E-05
14:96096022	T	C	0.91	0.94	0.64 [0.52-0.78]	2.28E-05
6:145025611[a]	T	C	0.92	0.89	1.51 [1.24-1.85]	2.65E-05
14:96091222	T	C	0.09	0.06	1.56 [1.27-1.91]	3.18E-05
5:2275109[a]	T	C	0.56	0.50	1.27 [1.13-1.42]	3.61E-05
2:219341618[a]	G	C	0.01	0.03	0.38 [0.22-0.64]	3.64E-05
19:8458145[a]	G	A	0.45	0.39	1.26 [1.13-1.41]	3.73E-05
9:13688437[a]	C	A	0.13	0.09	1.44 [1.22-1.71]	4.09E-05
1:228703236[a]	T	C	0.83	0.79	1.35 [1.16-1.56]	4.23E-05
3:12414420[a]	T	C	0.82	0.86	0.73 [0.63-0.85]	4.31E-05
14:96033565[a]	T	C	0.30	0.25	1.29 [1.15-1.46]	4.56E-05
5:178563002[a]	T	C	0.01	0.02	0.31 [0.16-0.60]	4.66E-05
18:25440258[a]	T	C	0.29	0.24	1.30 [1.15-1.47]	5.75E-05
17:48309874[a]	C	A	0.77	0.72	1.30 [1.14-1.48]	6.35E-05
10:100111392[a]	T	C	0.64	0.70	0.79 [0.70-0.88]	6.48E-05

1:228874049	T	C	0.25	0.30	0.77 [0.68-0.88]	6.63E-05
14:96025715	G	A	0.26	0.21	1.31 [1.15-1.49]	7.13E-05
17:40255743[a]	T	C	0.96	0.98	0.50 [0.36-0.69]	7.17E-05
20:34234620[a]	T	G	0.86	0.82	1.36 [1.16-1.59]	8.39E-05
20:34571846	T	C	0.85	0.81	1.35 [1.16-1.58]	8.55E-05
18:4470922[a]	G	A	0.85	0.80	1.34 [1.15-1.56]	8.83E-05
16:63119549[a]	T	C	0.46	0.41	1.25 [1.12-1.40]	8.98E-05
20:34285442	T	C	0.14	0.18	0.74 [0.63-0.86]	9.07E-05
14:96086814	G	A	0.09	0.06	1.50 [1.23-1.83]	9.16E-05
20:34432670	G	A	0.14	0.18	0.74 [0.63-0.86]	9.26E-05
20:34219496	T	C	0.86	0.82	1.36 [1.16-1.59]	9.36E-05
21:47654301	T	C	0.02	0.05	0.53 [0.38-0.75]	9.84E-05

[a] Independent variants. Data are displayed in GRCh37.p13, forward strand; *P* value has been calculated by Maximum Likelihood Ratio Test (MLRT). Abbreviations: Chr, chromosome; EA, effect allele; NEA, non-effect allele; EAF, effect allele frequency; OR, odds ratio; CI, confidence interval.

Supplementary Table 3. Summary of loci associated with DDH adjusted for gender in the discovery GWAS.

Chr: Position	EA	NEA	EAF Cases	EAF Controls	OR [95% CI]	*P* value
20:34025756	A	G	0.69	0.58	1.57 [1.30-1.77]	1.84E-14
20:34001058	T	C	0.71	0.62	1.50 [1.33-1.69]	1.43E-11
20:34025983	A	G	0.71	0.62	1.51 [1.33-1.70]	1.43E-11
20:33864484	G	A	0.33	0.43	0.67 [0.60-0.76]	2.16E-11
20:33952620	G	A	0.29	0.38	0.67 [0.59-0.76]	2.98E-11
20:33975181	C	A	0.29	0.38	0.67 [0.59-0.76]	2.98E-11
20:33971914	T	C	0.71	0.62	1.49 [1.32-1.68]	3.16E-11
20:34001250	G	A	0.29	0.38	0.67 [0.60-0.76]	4.04E-11
20:33894463	G	A	0.30	0.38	0.68 [0.61-0.77]	2.58E-10
20:33907161	T	C	0.70	0.62	1.46 [1.30-1.65]	2.84E-10
20:33914208	G	A	0.30	0.38	0.69 [0.61-0.77]	3.88E-10
20:34048161	T	C	0.18	0.24	0.70 [0.60-0.80]	2.30E-07
20:34059675	G	A	0.18	0.24	0.70 [0.61-0.80]	2.83E-07
20:34097353	T	C	0.82	0.76	1.43 [1.24-1.65]	2.84E-07
20:33909784	T	C	0.24	0.30	0.72 [0.63-0.82]	3.97E-07
20:33799280	T	G	0.64	0.57	1.33 [1.19-1.50]	6.48E-07
14:96035911	T	C	0.08	0.05	1.75 [1.41-2.19]	1.28E-06
12:1995403	T	C	0.14	0.10	1.50 [1.28-1.77]	1.83E-06
1:35208698	T	C	0.43	0.36	1.31 [1.17-1.46]	3.08E-06
20:34143092	G	A	0.18	0.23	0.73 [0.63-0.84]	6.25E-06
14:96085342	T	C	0.91	0.94	0.62 [0.51-0.76]	7.34E-06
20:33825378	G	A	0.87	0.82	1.42 [1.21-1.66]	1.09E-05
14:88651006	T	C	0.64	0.70	0.77 [0.69-0.87]	1.12E-05
1:85716807	G	A	0.84	0.8	1.38 [1.19-1.61]	1.15E-05
16:78430875	T	G	0.91	0.88	1.51 [1.24-1.83]	1.40E-05
20:34075998	T	C	0.87	0.82	1.41 [1.20-1.65]	1.72E-05
14:96096022	T	C	0.91	0.94	0.64 [0.52-0.78]	2.20E-05
6:145025611	T	C	0.92	0.89	1.51 [1.24-1.85]	2.66E-05
14:96091222	T	C	0.09	0.06	1.56 [1.27-1.91]	3.05E-05
5:2275109	T	C	0.56	0.50	1.27 [1.13-1.42]	3.41E-05
19:8458145	G	A	0.45	0.39	1.26 [1.13-1.41]	3.75E-05
9:13688437	C	A	0.13	0.09	1.44 [1.22-1.71]	3.78E-05
14:96033565	T	C	0.30	0.25	1.29 [1.15-1.46]	4.26E-05
3:12414420	T	C	0.82	0.86	0.73 [0.63-0.85]	4.33E-05
1:228703236	T	C	0.83	0.79	1.35 [1.16-1.56]	4.34E-05
18:25440258	T	C	0.29	0.24	1.30 [1.15-1.47]	6.07E-05
17:48309874	C	A	0.77	0.72	1.30 [1.14-1.48]	6.45E-05
1:228874049	T	C	0.25	0.30	0.77 [0.68-0.88]	6.78E-05
14:96025715	G	A	0.26	0.21	1.31 [1.15-1.49]	6.86E-05

10:100111392	T	C	0.64	0.70	0.79 [0.70-0.88]	6.94E-05
20:34234620	T	G	0.86	0.82	1.36 [1.16-1.59]	8.59E-05
14:96086814	G	A	0.09	0.06	1.50 [1.23-1.83]	8.70E-05
20:34571846	T	C	0.85	0.81	1.35 [1.16-1.58]	8.71E-05
16:63119549	T	C	0.46	0.41	1.25 [1.12-1.40]	9.18E-05
20:34285442	T	C	0.14	0.18	0.74 [0.63-0.86]	9.29E-05
18:4470922	G	A	0.85	0.80	1.34 [1.15-1.56]	9.41E-05
20:34432670	G	A	0.14	0.18	0.74 [0.63-0.86]	9.44E-05
20:34219496	T	C	0.86	0.82	1.36 [1.16-1.59]	9.61E-05
18:52253051	G	A	0.49	0.44	1.25 [1.12-1.40]	9.71E-05
14:34186045	G	A	0.73	0.78	0.77 [0.68-0.88]	9.88E-05

Data are displayed in GRCh37.p13, forward strand; *P* value has been calculated by Maximum Likelihood Ratio Test (MLRT). Abbreviations: Chr, chromosome; EA, effect allele; NEA, non-effect allele; EAF, effect allele frequency; OR, odds ratio; CI, confidence interval.

Supplementary Table 4. Summary of loci associated with DDH in the discovery, replication and meta-analysis stages.

			Discovery			Replication			Meta-analysis		
**Chr:Position**	**EA**	**NEA**	**OR[95% CI]**	***P* value**	**EAF**	**OR[95% CI]**	***P* value**	**EAF**	**OR[95% CI]**	***P* value**	**Effects**
1:35208698	T	C	1.31 [1.17-1.46]	3.10E-06	0.37	0.97 [0.88-1.07]	0.585483	0.37	1.10 [1.03-1.19]	0.008636	+-
1:85716807	G	A	1.38 [1.19-1.61]	1.15E-05	0.81	1.03 [0.92-1.16]	0.576298	0.81	1.15 [1.05-1.27]	0.002327	++
1:228703236	T	C	1.35 [1.16-1.56]	4.23E-05	0.79	1.09 [0.97-1.22]	0.154869	0.79	1.18 [1.08-1.29]	3.37E-04	++
2:133978555	G	A	0.81 [0.72-0.90]	0.000143	0.59	1.01 [0.92-1.11]	0.844429	0.59	0.92 [0.86-0.99]	0.022595	-+
3:12414420	T	C	0.73 [0.63-0.85]	4.31E-05	0.86	0.93 [0.82-1.05]	0.252138	0.85	0.84 [0.76-0.92]	3.24E-04	--
5:2275109	T	C	1.27 [1.13-1.42]	3.61E-05	0.52	1.02 [0.93-1.12]	0.667487	0.52	1.11 [1.04-1.20]	0.002734	++
5:178563002	T	C	0.31 [0.16-0.60]	4.66E-05	0.02	0.89 [0.62-1.28]	0.532669	0.02	0.70 [0.51-0.97]	0.030672	--
7:85031803	T	C	1.26 [1.12-1.42]	0.000122	0.32	1.05 [0.95-1.16]	0.302483	0.32	1.14 [1.05-1.22]	9.12E-04	++
7:142563253	T	G	4.74 [1.73-12.95]	0.000115	0.99	1.58 [0.93-2.69]	0.070095	0.99	2.01 [1.26-3.20]	0.003495	++
9:13688437	C	A	1.44 [1.22-1.71]	4.09E-05	0.10	1.20 [1.04-1.39]	0.017078	0.10	1.30 [1.16-1.45]	4.44E-06	++
10:100111392	T	C	0.79 [0.70-0.88]	6.48E-05	0.69	0.88 [0.80-0.97]	0.008958	0.68	0.84 [0.78-0.90]	4.38E-06	--
12:1995403	T	C	1.50 [1.28-1.77]	1.73E-06	0.11	1.03 [0.89-1.20]	0.658354	0.11	1.22 [1.10-1.36]	3.17E-04	++
14:34184786a	G	A	0.80 [0.70-0.90]	0.000342	0.75	0.98 [0.88-1.09]	0.683176	0.74	0.90 [0.83-0.97]	0.00781	--
14:88646827b	T	C	0.88 [0.78-0.99]	0.03146	0.68	1.05 [0.95-1.15]	0.386302	0.68	0.97 [0.90-1.05]	0.464382	-+
14:96033565	T	C	1.29 [1.15-1.46]	4.56E-05	0.26	1.01 [0.91-1.13]	0.804449	0.25	1.13 [1.04-1.22]	0.003681	++
14:96035911	T	C	1.75 [1.41-2.19]	1.34E-06	0.05	1.10 [0.88-1.37]	0.426304	0.04	1.39 [1.19-1.63]	3.73E-05	++
16:63119549	T	C	1.25 [1.12-1.40]	8.98E-05	0.42	0.94 [0.86-1.04]	0.228532	0.41	1.06 [0.99-1.14]	0.098933	+-
16:78430875	T	G	1.51 [1.24-1.83]	1.45E-05	0.88	0.90 [0.78-1.03]	0.129695	0.89	1.08 [0.96-1.21]	0.206753	+-
17:40255743	T	C	0.50 [0.36-0.69]	7.17E-05	0.98	0.95 [0.70-1.30]	0.74986	0.98	0.70 [0.56-0.87]	0.001723	--
17:48309874	C	A	1.30 [1.14-1.48]	6.35E-05	0.73	1.02 [0.92-1.13]	0.721746	0.73	1.12 [1.03-1.22]	0.005983	++
18:4470922	G	A	1.34 [1.15-1.56]	8.83E-05	0.81	0.90 [0.80-1.02]	0.095146	0.82	1.05 [0.96-1.16]	0.282519	+-
18:25417967c	T	C	0.81 [0.72-0.92]	0.000879	0.74	1.00 [0.90-1.11]	0.936517	0.75	0.91 [0.84-0.99]	0.024394	--
19:8458145	G	A	1.26 [1.13-1.41]	3.73E-05	0.40	0.99 [0.91-1.09]	0.909271	0.40	1.10 [1.02-1.18]	0.010679	+-
20:33825378	G	A	1.42 [1.21-1.66]	1.09E-05	0.83	1.27 [1.12-1.45]	0.000185	0.83	1.33 [1.20-1.47]	3.18E-08	++

20:34025756	A	G	1.57 [1.30-1.77]	1.72E-14	0.61	1.37[1.24-1.51]	1.33E-10	0.61	1.44 [1.34-1.56]	3.55E-22	--
20:34294409	G	A	1.41 [1.18-1.69]	0.000105	0.87	1.41 [1.20-1.64]	8.01E-06	0.88	1.41 [1.25-1.58]	1.15E-08	++
21:47590975	T	C	1.30 [1.14-1.50]	0.000126	0.77	1.02 [0.91-1.13]	0.771215	0.77	1.12 [1.03-1.22]	0.009687	++
21:47614553	G	A	1.96 [1.41-2.72]	1.20E-05	0.95	0.91 [0.74-1.12]	0.362843	0.95	1.13 [0.95-1.35]	0.169713	+-

[a]proxy of 14:34186045(r^2=0.70); [b]proxy of 14:88651006 (r^2=0.54); [c]proxy of 18:25440258 (r^2=0.86); *P* value has been calculated by Maximum Likelihood Ratio Test (MLRT); Abbreviations: Chr, chromosome; EA, effect allele; NEA, non-effect allele; EAF, effect allele frequency; OR, odds ratio; CI, confidence interval.

Supplementary Table 5. LD calculations between the 3 variants in chromosome 20 that showed association with DDH at genome-wide significance.

SNP_A	SNP_B	R2
rs12479765	rs2050729	0.029
rs143384	rs12479765	0.154
rs143384	rs2050729	0.121

Supplementary Table 6. Linkage disequilibrium (LD) calculations of the variants between the significant genes of the gene-based analysis.

SNP_A	GENE	SNP_B	GENE	R2[a]
20:33864484	MMP24	20:33894463	UQCC1	0.384754
20:33864484	MMP24	20:33907161	UQCC1	0.385332
20:33864484	MMP24	20:33909784	UQCC1	0.260634
20:33864484	MMP24	20:33914208	UQCC1	0.385541

[a] pairs with r2 values less than 0.2 are not included in the table.

Supplementary Table 7. Single-tissue expression quantitative trait loci (eQTLs) associations between rs143384 and multiple genes across various tissues in order of significance.

Gene Symbol	SNP ID	*P* Value	Effect Size	Tissue
UQCC1	rs143384	5.20E-19	-0.42	Cells - Transformed fibroblasts
UQCC1	rs143384	3.60E-09	-0.19	Muscle – Skeletal
UQCC1	rs143384	4.30E-09	-0.26	Lung
UQCC1	rs143384	1.80E-07	-0.23	Esophagus – Mucosa
UQCC1	rs143384	2.70E-07	-0.27	Nerve – Tibial
RPL36P4	rs143384	5.80E-07	0.41	Adipose – Subcutaneous
UQCC1	rs143384	6.30E-07	-0.22	Whole Blood
RPL36P4	rs143384	8.60E-07	0.4	Cells - Transformed fibroblasts
FAM83C	rs143384	0.0000015	-0.19	Skin - Sun Exposed (Lower leg)
UQCC1	rs143384	0.0000018	-0.24	Skin - Sun Exposed (Lower leg)
RP3-477O4.16	rs143384	0.0000022	0.62	Testis
FAM83C	rs143384	0.0000045	-0.26	Esophagus – Mucosa
UQCC1	rs143384	0.0000068	-0.17	Artery – Tibial
RP3-477O4.16	rs143384	0.0000085	0.42	Nerve – Tibial
UQCC1	rs143384	0.000013	-0.23	Adipose – Subcutaneous
CEP250	rs143384	0.000016	-0.49	Brain – Cortex
GDF5	rs143384	0.000017	-0.28	Lung
GDF5	rs143384	0.000026	0.43	Pituitary
MAP1LC3A	rs143384	0.000029	0.23	Stomach
RPL36P4	rs143384	0.00003	0.38	Esophagus – Mucosa
MAP1LC3A	rs143384	0.000031	0.16	Artery – Tibial
CEP250	rs143384	0.000031	-0.23	Muscle – Skeletal
PROCR	rs143384	0.000035	0.54	Cells - EBV-transformed lymphocytes
MAP1LC3A	rs143384	0.000039	0.2	Esophagus – Muscularis
RPL36P4	rs143384	0.000043	0.35	Skin - Sun Exposed (Lower leg)
RPL36P4	rs143384	0.000045	0.37	Nerve – Tibial
RP3-477O4.16	rs143384	0.000062	0.56	Brain – Cerebellum
RPL36P4	rs143384	0.000067	0.56	Brain - Nucleus accumbens (basal ganglia)
UQCC1	rs143384	0.00007	-0.21	Adipose - Visceral (Omentum)
RPL36P4	rs143384	0.000073	0.31	Whole Blood
CPNE1	rs143384	0.000087	-0.24	Lung
RPL36P4	rs143384	0.00009	0.35	Thyroid
EIF6	rs143384	0.00014	0.15	Skin - Sun Exposed (Lower leg)
CEP250	rs143384	0.00015	-0.36	Brain – Cerebellum
GDF5	rs143384	0.00018	0.28	Esophagus – Muscularis
EIF6	rs143384	0.0002	-0.12	Cells - Transformed fibroblasts
CEP250	rs143384	0.00023	0.19	Esophagus – Mucosa

Data Source: GTEx Analysis Release V6p (dbGaP Accession phs000424.v6.p1)

Supplementary Table 8. Self-reported DDH history questionnaire.

**Q1. Did you have any problems with your hip as a child?**	**No**	**Yes**	
**Q2. Have you ever had a dislocated hip as a child?**	**No**	**Yes**	
**If so, which side?**	**Right**	**Left**	**Both**
**Q3. Were you ever treated with a harness or hip cast as a child?**	**No**	**Yes**	
**Q4. Were you ever treated with leg traction as a child?**	**No**	**Yes**	
**Q5. Did you have any operations on your hip as a child?**	**No**	**Yes**	
**Q6. Have you ever been told you've suffered from any of the following:**			
**Perthes' disease**	**No**	**Yes**	
**Slipped Capital Femoral Epiphysis**	**No**	**Yes**	
**Congenital Femoral Deficiency**	**No**	**Yes**	
**Septic Arthritis (Hip Infection)**	**No**	**Yes**	
**Q7. Do you have any of the following medical conditions:**			
**Cerebral Palsy**	**No**	**Yes**	
**Spina Bifida**	**No**	**Yes**	
**Arthrogryposis**	**No**	**Yes**	
**Larsen's Syndrome**	**No**	**Yes**	
**Q8. At what age did you start having problems with your hip?**			
**Birth - 4 years 5 - 10 years 10 - 15 years 15 - 20 years**			
**Q9. Has anyone in your family had hip operations as a child?**	**No**	**Yes**	
**If Yes, who?** ***Please mark all those that apply:***			
**Mother Father Brother Sister**			
**Q10. Have you ever sustained a fracture of your hip or pelvis?**	**No**	**Yes**	

Supplementary Table 9. Quality control procedure.

**QC criterion**	**DDH cases**	**UKHLS controls**
Call rate	10	56
Sex-mismatch	17	55
Heterozygosity rate	11	148
Relatedness (duplicates)	10	7
Ethnic outliers	29	340
Identity checks	15	3
Samples with no record	0	65
Radiographic validation (for cases)	9	NA
Total exclusions	64	519
Total samples after QC	770	9961
Total samples included in the analysis	770	3364

Supplementary Table 10. Replication results by excluding 66 NJR subjects that failed genome-wide genotyping QC.

			Replication by excluding 66 NJR subjects		
**Chr:Position**	**EA**	**NEA**	**OR[95% CI]**	***P* value**	**EAF**
1:35208698	T	C	0.97 [0.88-1.07]	0.575548	0.37
1:85716807	G	A	1.08 [0.95-1.22]	0.230944	0.81
1:228703236	T	C	1.08 [0.96-1.21]	0.211932	0.79
2:133978555	G	A	1.01 [0.92-1.11]	0.860789	0.59
3:12414420	T	C	0.92 [0.81-1.05]	0.213183	0.85
5:2275109	T	C	1.02 [0.92-1.12]	0.752313	0.52
5:178563002	T	C	0.80 [0.55-1.18]	0.248259	0.02
7:85031803	T	C	1.06 [0.96-1.17]	0.268066	0.32
7:142563253	T	G	1.53 [0.88-2.64]	0.109704	0.99
9:13688437	C	A	1.20 [1.03-1.40]	0.022074	0.10
10:100111392	T	C	0.87 [0.79-0.97]	0.009538	0.68
12:1995403	T	C	1.05 [0.90-1.22]	0.522351	0.11
14:34184786a	G	A	1.00 [0.89-1.11]	0.940591	0.74
14:88646827b	T	C	1.06 [0.96-1.17]	0.278372	0.68
14:96033565	T	C	1.01 [0.90-1.12]	0.88546	0.25
14:96035911	T	C	1.10 [0.87-1.39]	0.428018	0.04
16:63119549	T	C	0.94 [0.86-1.04]	0.243315	0.41
16:78430875	T	G	0.89 [0.77-1.03]	0.124158	0.89
17:40255743	T	C	1.05 [0.75-1.46]	0.786826	0.98
17:48309874	C	A	1.00 [0.90-1.12]	0.939857	0.73
18:4470922	G	A	0.92 [0.81-1.04]	0.178533	0.82
18:25417967c	T	C	1.00 [0.89-1.11]	0.973642	0.75
19:8458145	G	A	1.00 [0.91-1.10]	0.978497	0.40
20:33825378	G	A	1.31 [1.14-1.49]	6.41E-05	0.83
20:34025756	A	G	1.37 [1.24-1.52]	3.48E-10	0.61
20:34294409	G	A	1.46 [1.24-1.71]	2.07E-06	0.88
21:47590975	T	C	1.01 [0.90-1.13]	0.856541	0.77
21:47614553	G	A	0.89 [0.72-1.11]	0.30588	0.95

[a]proxy of 14:34186045(r^2=0.70); [b]proxy of 14:88651006 (r^2=0.54); [c]proxy of 18:25440258 (r^2=0.86); *P* value has been calculated by Maximum Likelihood Ratio Test (MLRT); Abbreviations: Chr, chromosome; EA, effect allele; NEA, non-effect allele; EAF, effect allele frequency; OR, odds ratio; CI, confidence interval.
