# Supplementary material for: Genome-wide association study of developmental dysplasia of the hip identifies an association with GDF5
Source: Commun Biol. 2018 May 31;1:56. doi: 10.1038/s42003-018-0052-4 (PMC6123669; doi:10.1038/s42003-018-0052-4)
Supplement: Supplementary file 2 — Description of Additional Supplementary Files [file 42003_2018_52_MOESM2_ESM.docx]

**Description of Additional Supplementary Files**

File Name: Supplementary Data 1

Description: Excel file showing variants that are expression quantitative trait loci (eQTL) that regulate nearby genes modulating expression in various tissues.

File Name: Supplementary Data 2

Description: Excel file showing results of LDHub analysis exploring the genetic correlation between DDH and 235 other traits and diseases.
